# A Systematic Review of the Cost-Effectiveness of Cleft Care in Low- and Middle-Income Countries: What is Needed?

**DOI:** 10.1177/10556656221111028

**Published:** 2022-07-03

**Authors:** Karen Y. Chung, George Ho, Aysegul Erman, Joanna M. Bielecki, Christopher R. Forrest, Beate Sander

**Affiliations:** 1 7938 Division of Plastic Surgery, Department of Surgery, University of Toronto, Toronto, ON, Canada; 2 Toronto Health Economics and Technology Assessment (THETA) collaborative, 7989 University Health Network, Toronto, ON, Canada; 3 Toronto Health Economics and Technology Assessment (THETA), 7938 University of Toronto, 7989 University Health Network, Toronto, ON, Canada; 4 Institute of Health Policy, Management and Evaluation (IHPME), 7938 University of Toronto, Toronto, ON, Canada; 5 153300 Public Health Ontario, Toronto, ON, Canada; 6 50010 ICES, Toronto, ON, Canada

**Keywords:** cleft, cost-effectiveness, global surgery

## Abstract

**Objective:**

The objective of this paper is to conduct a systematic review that summarizes the cost-effectiveness of cleft lip and/or palate (CL/P) care in low- and middle-income countries (LMICs) based on existing literature.

**Design:**

We searched eleven electronic databases for articles from January 1, 2000 to December 29, 2020. This study is registered in PROSPERO (CRD42020148402). Two reviewers independently conducted primary and secondary screening, and data extraction.

**Setting:**

All CL/P cost-effectiveness analyses in LMIC settings.

**Patients, Participants:**

In total, 2883 citations were screened. Eleven articles encompassing 1,001,675 patients from 86 LMICs were included.

**Main Outcome Measures:**

We used cost-effectiveness thresholds of 1% to 51% of a country's gross domestic product per capita (GDP/capita), a conservative threshold recommended for LMICs. Quality appraisal was conducted using the Joanna Briggs Institute (JBI) checklist.

**Results:**

Primary CL/P repair was cost-effective at the threshold of 51% of a country's GDP/capita across all studies. However, only 1 study met at least 70% of the JBI criteria. There is a need for context-specific cost and health outcome data for primary CL/P repair, complications, and existing multidisciplinary management in LMICs.

**Conclusions:**

Existing economic evaluations suggest primary CL/P repair is cost-effective, however context-specific local data will make future cost-effectiveness analyses more relevant to local decision-makers and lead to better-informed resource allocation decisions in LMICs.

## Introduction

Cleft lip and/or palate (CL/P) is the most common craniofacial congenital anomaly worldwide. Yet, CL/P is undertreated in low- and middle-income countries (LMICs). Untreated children with CL/P experience malnutrition, poor dentition, ear infections, speech deficiencies, and extreme social stigma which has resulted in abandonment or infanticide.^1–4^ These experiences are exacerbated by delays in care.

Economic evaluations help clinicians and policymakers make informed decisions in resource-constrained settings.^5^ Cost-effectiveness analyses are used to compare the value of interventions, and prioritized interventions in LMICs are those that are cost-effective and feasible.^5^ The objective of this paper is to conduct a systematic review that summarizes the cost-effectiveness of CL/P care in LMICs based on existing literature. This will consolidate existing decision-analyses for CL/P in LMICs and assess the methodologic quality of these analyses in how they can be used to help inform LMIC resource allocation decisions.

## Methods

**Figure 1.**
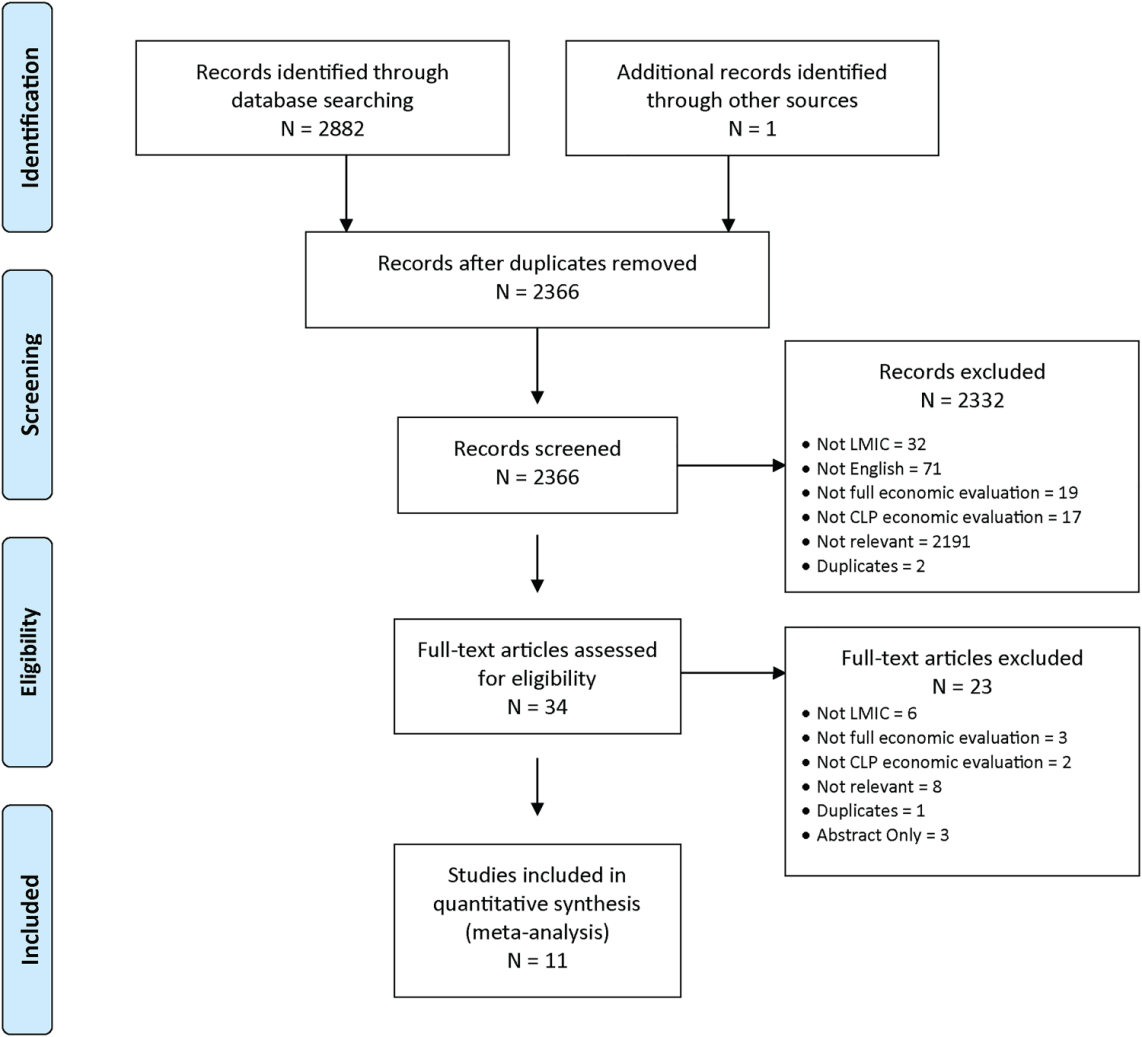
Preferred reporting items for systematic reviews and meta-analysis (PRISMA) flow diagram.

This systematic review is conducted in accordance with Preferred Reporting Items for Systematic Reviews and Meta-Analyses (PRISMA) guidelines (Figure 1). The full search strategy with key terms and protocol is registered and available on PROSPERO, number CRD42020148402 (Appendix 1). The search strategy was designed and executed by an Information Specialist (JB) with expertise in systematic reviews with additional clinical input (KC).

We searched 11 electronic databases from January 1, 2000 to December 29, 2020 using keywords and medical subject headings related to the following concepts: cleft lip, cleft palate, cost-effectiveness, and economic evaluations. The search strategy was initially developed in Ovid MEDLINE, and then adapted to the syntax and subject headings of the other databases: Global Health Cost-Effectiveness Analysis Registry, Cost-Effectiveness Analysis (CEA) Registry, Ovid EMBASE, Global Index Medicus, ScHARRHUD database, Cochrane Database of Systematic Reviews database, and the Center for Reviews and Dissemination Databases, which includes the Health Technology Assessment Database (HTA), HTA Database Canadian Repository, the NHS Economic Evaluation Database (NHS EED) and the Database of Abstracts of Reviews of Effects. Additionally, we hand-searched gray literature and bibliographies of identified publications. Finally, we searched PROSPERO for ongoing or recently completed systematic reviews.

Two reviewers (KC, GH) independently assessed the titles, abstracts, and full texts for inclusion using DistillerSR. Full economic evaluations that reported cost-benefit analyses, cost-effectiveness analyses, or cost-utility analyses of primary cleft lip and/or palate repair in LMIC settings as defined by the World Bank were included.^6^ Only English language studies were included due to feasibility and resource constraints. We excluded studies that were not full economic evaluations, such as studies that reported only health outcomes, or cost-minimization, cost-consequence and cost-of-illness studies.

Two reviewers (KC and AE or GH) independently extracted data and conducted the quality appraisal using the Joanna Briggs Institute (JBI) critical appraisal tool for economic evaluations.^7^ These criteria are a validated international tool to evaluate evidence related to the feasibility, appropriateness, meaningfulness, and effectiveness of health care interventions.^7^

### Analysis

We reported costs in 2020 International Dollars (USD). Where country-specific costs were provided, these were inflated to the most recent year based on the country's consumer price index.^6^ For articles that provided summaries across countries and regions, we used the inflation rate for the specific region or the region with the highest volume of cleft surgeries, as appropriate. Cost-effectiveness analyses are composed of cost-utility analyses or cost-benefit analyses. Cost-utility analysis is a method of economic evaluation, where health outcomes are valued using a generic measure of health, such as Disability-Adjusted Life Years (DALYs).^8^ The results are reported as an Incremental Cost-Effectiveness Ratio of dollars per DALY averted. In cost-benefit analyses, health outcomes are valued in monetary terms and can be based on the Gross National Index per capita (GNI/capita) for each country.^5,9^ Surgeries performed in a country with a higher GNI/capita will thus report a higher economic benefit compared to surgeries performed in a country with a lower GNI/capita. The results are reported as a ratio of costs to benefits or as a net benefit or loss, and if they were based on disability weights from the Global Burden of Disease (GBD) study then these values may be adjusted by age-weighting at 4% and discounting at 3%.^9,10^ We assessed the cost-effectiveness of primary CL/P repair using cost-effectiveness thresholds of 1% to 51% of a country's gross domestic product per capita (GDP/capita), a conservative estimate of health spending recommended for LMICs.^11–13^

## Results

We screened 2883 citations and included 11 economic evaluations. The 11 studies encompassed a total of 1,001,675 patients from 86 countries.^10,14–24^ Four studies considered patients from a low-income country (Table 1). There were no studies that considered multidisciplinary care. None of the included studies used a model-based economic evaluation. All studies were based on a lifetime horizon. All costs came from an NGO perspective and were reported in international USD. Eight of the 11 studies conducted a cost-utility analysis using DALYs to value health outcomes, with 6 studies conducting cost-benefit analyses.

**Table 1.** Study Demographics.

Citation	NGO	Country or Countries	Number of patients	Number of procedures	Demographics (age, gender if reported)	Patient cleft type
Magee et al. (2010)^19^	Operation Smile	Vietnam, Kenya, Russia, Nicaragua	303	Cleft lip: 133 Cleft palate: 170	Age less than 5 years: 72% Range: 142 days to 41 years Male to female ratio: Not listed	Cleft lip: Yes Cleft palate: Yes Cleft lip and palate: Not listed Complications: Not listed
Corlew (2010)^14^	Interplast (now ReSurge)	Nepal	568	Cleft lip: 402 Cleft palate: 166	Younger than 1-28 years Female: 198 Male: 370	Cleft lip: Yes Cleft palate: Yes Cleft lip and palate: Yes (4 Patients) Complications: Not listed
Alkire et al. (2011)^10^	N/A	SSA	34,683 (from 2008 U.S. African American cleft incidence and SSA population)	Cleft lip: 18,918 Cleft palate: 15,765	N/A	Cleft lip: Yes Cleft palate: Yes Cleft lip and palate: Not listed Complications: Not listed
Moon et al. (2012)^20^	Smile for Children	Vietnam	2907	Cleft lip: 845 Cleft palate: 735	Average age: 8.3 years Male to female ratio: Not listed	Cleft Lip: Yes Cleft Palate: Yes Cleft Lip and Palate: Not listed Complications: Not listed
Hughes et al. (2012)^18^	Hands Across the World	Ecuador	Cleft lip and palate: 123 Cleft lip: 17 Cleft palate: 38	Cleft lip: 48 Cleft palate: 54	Not listed	Cleft lip: Yes Cleft palate: Yes Cleft lip and palate: Yes Complications: Not listed
Poenaru (2013)^22^	Smile Train	79 Countries	364,467	536,846	Mean Age: 6.2 years (2.2 years: 9.8 years) Male to female ratio: Not listed	Cleft lip: Yes Cleft palate: Yes Cleft lip and palate: Not listed Complications: Yes
Rattray et al. (2013)^24^	Children's Surgical Center	Cambodia	17	17	Not listed	Cleft lip: Yes Cleft palate: Yes Cleft lip and palate: Not listed Complications: Not listed
Hackenberg et al. (2015)^16^	Operation Smile, Guwahati Cleft Care Center	India	Center: 4930 Missions: 7358	Cleft lip: 4511 Cleft palate: 1615 Cleft lip and palate: 155	Missions age (Mean, SD): 11.9 (±11.2) Center age (Mean, SD): 11.8 (±11.6) Missions (Male:Female) 1.27:1 Care center (Male: Female) 1:1	Cleft lip: Yes Cleft palate: Yes Cleft lip and palate: Yes Complications: Not listed
Poenaru et al. (2016)^23^	Smile Train	83 Countries	548,147	Cleft lip: 58% Cleft palate: 42%	Not listed	Cleft lip: Yes Cleft palate: Yes Cleft lip and palate: Not listed Complications: Not listed
Hamze et al. (2017)^17^	Amref Health Africa and Smile Train	Burundi, Democratic Republic of Congo, Ethiopia, Kenya, Rwanda, South Sudan, Tanzania, Uganda	37,274	37,274	Median age: 5.38 years Male: 62% Female: 38%	Cleft lip: Yes Cleft palate: Yes Cleft lip and palate: Yes (3%) Complications: Yes, considered same as CL
Nandoskar et al. (2020)^21^	Royal Australasian College of Surgeons	Indonesia	843	843	Cleft lip age range: 0-52 Cleft palate age range: 0-37	Cleft lip: Yes Cleft palate: Yes Cleft lip and palate: Not listed Complications: Not listed

Abbreviations: SSA, Subsaharan Africa; NGO, Non-Governmental Organization.

### Data Inputs

All studies used cost data from NGOs: Operation Smile (n = 3), Interplast (n = 2), Smile Train (n = 3), Smile for Children (n = 1), the Children's Surgical Center (n = 1), and Hands Across the World (n = 1). Age at surgery ranged from 142 days old to 74 years old. DALYs were derived from isolated cleft lip or isolated cleft palate disability weights produced by the GBD studies in 1990 or 2004 for ten of the 11 studies. The remaining study measured and applied disability weights based on the expertise of 5 surgeons.^24^ Although studies may have included patients with combined cleft lip and cleft palate, ten of the studies did not consider a disability weight for untreated cleft lip and palate, and 1 study used isolated cleft palate as a proxy for these patients.^17^

The health impact of CL/P complications, i.e. fistula or lip/nose revision, was included in 2 studies. One study cited a disability weight of 0.05 for both the palatine fistula and the lip revision.^22^ The other study used isolated cleft lip as a proxy for complications and included this in the DALYs averted for CL/P.^17^

### Costs

All studies reported costs in international dollars (USD). Costs regarding whether NGOs used a center- or mission-based approach are outlined in Table 2. Eight articles provided an outline of cost components. Major cost components discussed in the articles were: staff salary and recruitment (88%), medical supplies (75%), staff accommodation (50%), staff transportation (50%), NGO overhead (50%), patient transportation (50%), patient hospital costs (50%), and patient food (50%). No articles reported patient or caregiver wages lost or time loss because all costs were reported from the NGO perspective. Finally, no article considered the costs associated with untreated cleft lip and/or palate.

**Table 2.** Summary of Costs per Procedure, DALYs Averted, Cost per DALY Averted, and Cost-Effectiveness Thresholds.

Article	NGO (Center vs mission based)	Country(ies)/Year	Cost per cleft lip and cleft palate procedure (USD, 2020)	Cleft lip, cleft palate or both*	DALYs averted per patient (with 3% discounting, 2020)	Cost per DALY averted (USD, 2020)	World Health Organization threshold (1 times the GDP per capita)	51% GDP per capita cost-effectiveness threshold	1% GDP per capita cost-effectiveness threshold
Alkire et al. (2011)^10^	(Not specified)	Subsaharan Africa, 2008	316.27	Both	5.35	59.13	1572.61	801.72	8.02
Corlew (2010)^14^	Interplast, now ReSurge (Center-Based)	Nepal, 2005	513.40	Cleft lip	4.04	126.96	1025.80	523.16	5.23
				Cleft palate	10.59	48.48			
Magee et al. (2010)^19^	Operation Smile (Mission-Based)	Vietnam,2008	850.40	Both	8.18	100.32	2563.81	1307.54	13.08
		Nicaragua, 2008	816.03	Both	6.70	85.42	2028.90	1034.74	10.35
		Kenya, 2008	658.37	Both	3.16	146.30	1710.51	872.36	8.72
		Russia, 2008	685.40	Both	9.46	50.84	11,888.72	6063.25	60.63
Moon et al. (2012)^20^	Smile for Children (Mission-Based)	Vietnam, 2007	511.38	Both	Not listed	96.63	2563.81	1307.54	25.64
		Vietnam, 2008	519.39	Both	Not listed	113.35			
		Vietnam, 2009	366.73	Both	Not listed	70.37			
		Vietnam, 2010	426.33	Both	Not listed	89.26			
Hughes et al. (2012)^18^	Hands Across the World (Mission-based)	Ecuador, 2011	Not listed	Both	8.3	Not Listed			
Poenaru (2013)^22^	Smile Train (Center-based)	79 Countries, 2010	482.54	Both	4.32	111.81	1905.77	971.94	19.06
Rattray et al. (2013)^24^	Children's Surgical center (Center-based)	Cambodia, 2012	Not listed	Not listed	Not listed	102.67	1512.13	771.18	7.71
Hackenberg et al. (2015)^16^	Operation Smile Guwahati Cleft Care Missions (Both)	India, Medical Missions, 2012	1421.93	Both	4.74	239.21	2015.59	1027.95	10.28
		India, Care Center, 2012	2324.70	Both	4.74	440.20			
Poenaru et al. (2016)^23^	Smile Train (Center-Based)	83 Countries	443.89	Both	2.49	178.48	1905.77	971.94	9.72
Hamze et al. (2017)^17^	Smile Train and AMREF (Center-Based)	Eastern and Central Africa, 2014	400.25	Cleft lip	4.19	95.58	1572.61	802.03	15.73
				Cleft palate	10.40	38.48			
				Both	4.67	85.65			
Nandoskar et al. (2020)^21^	Royal Australasian College of Surgeons (Mission-based)	Indonesia, 2017	Not listed	Both	Not listed	704.6	4287.19	2186.47	42.87

*Both refers to both patients with isolated cleft lip and patients with isolated cleft palate. Abbreviations: GDP, gross domestic product; DALY, disability-adjusted life year, NGO, Non-Governmental Organization.

### Outcomes

Ten of the 11 articles valued health outcomes in terms of DALYs. DALYs averted per cleft lip or palate repair ranged from 1.2 to 10.7, dependent on the country (Table 2). The cost for primary CL/P repair ranged from $33.7 to 401.8, depending on the country and NGO (Table 2). The cost per DALY associated with surgery demonstrated that primary CL/P repair was cost-effective at the threshold of 51% of GDP per capita for all studies (Table 2). Only 1 study compared CL/P repair in relation to other interventions in Vietnam and found it to be cost-effective in comparison to human immunodeficiency virus/acquired immunodeficiency syndrome or tuberculosis treatment.^20^

Six cost-benefit analyses were reported, all of which used the human capital approach (Table 3). Using the human capital approach, the estimated economic benefit in USD for individual primary cleft lip repair ranged from $3191 (Indonesia) to $32,203 (Ecuador). The estimated economic benefit from individual cleft palate repair ranged from $4110 (Indonesia) to $87,393 (Ecuador).

**Table 3.** Summary of Estimated Individual and Population Economic Benefit Using the Value of a Statistical Life Approach and Human Capital Approach.

Individual economic benefit of cleft lip and cleft palate repair (2020 USD with 3% discounting)			
		Cleft lip	Cleft palate
Indonesia	VSL Income Elasticity 1.0	4809.3	6243.8
	VSL Income Elasticity 1.5	878.7	1140.8
	Human Capital Approach	3191.0	4110.2
Eastern and Central Africa			
VSL Income Elasticity 1.0		58,948.9	173,165.9
VSL Income Elasticity 1.5		9775.0	29,889.4
Human Capital Approach*		4240.7	12,339.3
Ecuador			
VSL Income Elasticity 1.0		187,396.2	515,842.9
VSL Income Elasticity 1.5		70,031.1	192,773.6
Human Capital Approach*		32,203.4	87,393.8
Nepal			
	VSL Income Elasticity 1.0	106,262.8	284,465.3
	Human Capital Approach*	4891.3	13,092.7
			
South Asia			
	Human Capital Approach	15,656.9	46,671.9
Sub-Saharan Africa	VSL Income Elasticity 0.55	112,704.0	296,882.2
	Human Capital Approach*	5269.4	13,874.4

*Age-weighting, as used in the Global Burden of Disease Study, is included.

Estimates of income elasticity of the value of a statistical life range can from 0.55 to 1.5 with evidence suggesting that larger income elasticity estimates are more appropriate when income is lower.^25^

### Quality Appraisal

**Figure 2.**
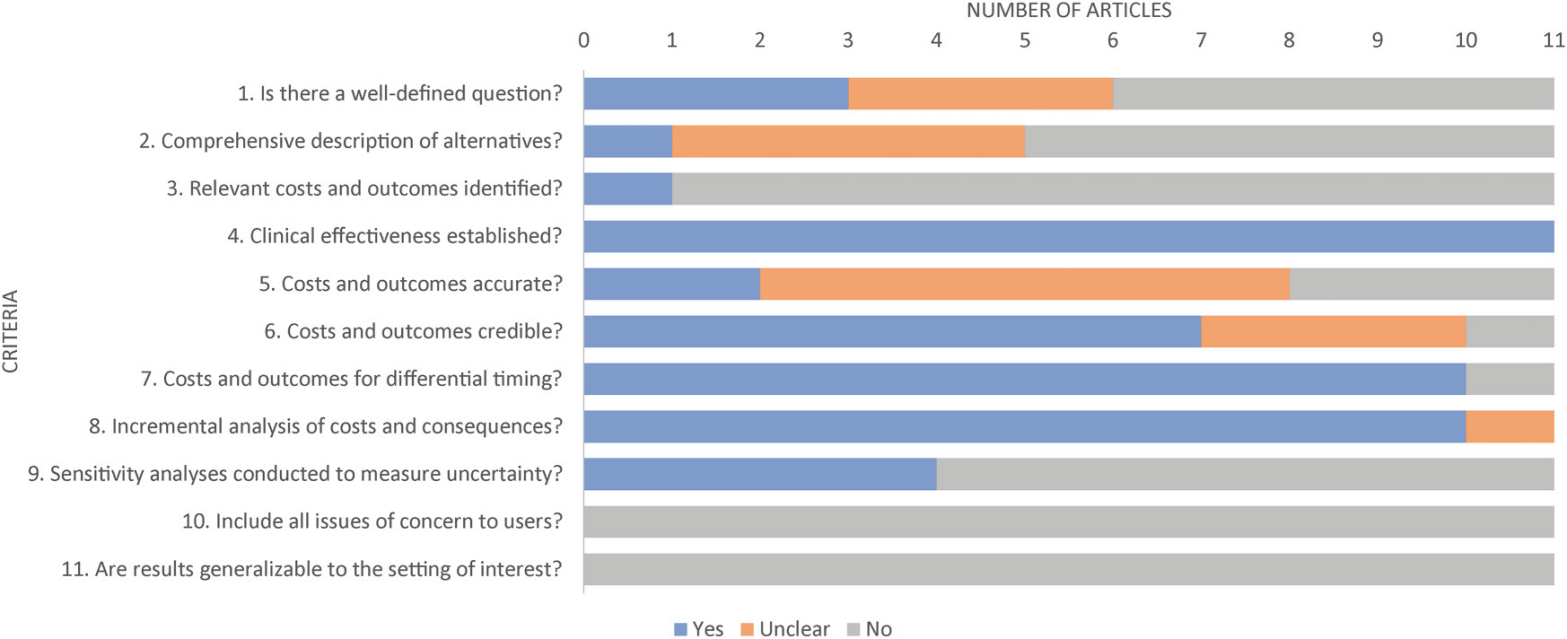
Joanna Briggs Institute (JBI) critical appraisal checklist for economic evaluations.

All articles met at least 1 of the 11 JBI criteria (Figure 2). However, only 1 study met at least 70% of JBI criteria. Nine of the 11 articles did not describe subsequent complications from primary CL/P repair or consider costs for patients without primary repair. Further, 10 of the 11 articles did not consider disability weights for patients with untreated combined cleft lip and cleft palate which can be considered more severe than isolated cleft types. Furthermore, of the studies that accounted for the costs, none of the studies considered wages lost from the patient and caregiver perspective during care received. None of the studies used health outcomes from patient or societal populations in LMICs. Finally, multidisciplinary care was not considered. The data inputs for the economic evaluations make the results less relevant to local-decision makers and not generalizable to the affected patients in LMICs.

## Discussion

This systematic review synthesizes the evidence on the economic value of CL/P repair in LMICs to date. All studies reported the costs of CL/P repair from the health care payer perspective, where the health care payer was the NGO. Local governments can assume the role of the health care payer and consider whether CL/P repair is cost-effective for their country. A cost-effectiveness threshold of up to 51% of the GDP/capita is recommended for LMICs.^11–13^ Based on this threshold, the cost per DALY for primary CL/P repair was cost-effective across all studies. However, several concerns were identified that could limit this implication.

First, it is suspected that GBD disability weights underestimate the disease severity and impact of treatment. Disability weights are considered ‘universal’; however, they came from international societal participants, a sample heavily skewed to those from high-income countries (HICs).^26–28^ Further, GBD descriptions do not include difficulties swallowing, malnutrition, and the extreme social stigma experienced by patients with CL/P in LMICs.^26^ Finally, disability weights for surgically treated CL/P were last estimated in 1990, by a small panel of elite international health experts and there is no consideration of multidisciplinary management.^26^ Second, all studies reported costs from the NGO perspective and thus the costs from the government perspective are unknown. Further, none of the studies considered opportunity costs for patients and their caregivers in receiving treatment. These context-specific costs can better inform cost-effectiveness analyses in these settings. Third, 10 of the 11 papers did not consider disability weights for untreated patients with combined cleft lip and palate. Cleft lip and palate is considered more severe than isolated cleft lip or cleft palate, and studies that omit this may further underestimate the burden of disease and cost-effectiveness of CL/P repair. Fourth, there was limited consideration of complications which may result from surgery, and finally, none of the articles considered multidisciplinary management.

Due to these concerns, existing CL/P cost-effectiveness analyses for LMICs may have underestimated the severity of CL/P and the impact of treatment. The main strength of this study is that it synthesizes existing evidence to highlight what is needed to better represent the cost-effectiveness of CL/P care and inform subsequent resource allocation decisions in LMICs. The main limitation of this systematic review is that we excluded 71 articles that were not published in English. However, none of these studies appeared to be relevant for CL/P economic evaluations when these articles were screened using an online translation tool.

Thanks to efforts from the Lancet Commission of Global Surgery, the 68th World Health Assembly passed the resolution on including surgery as a component of Universal Health Coverage.^29^ Thus, for policy decision-makers to make informed decisions in allocating public funds, cost-effective analyses are critical. The lack of context-specific health and cost data reported from LMICs is a limitation seen with existing economic evaluations for other surgical diseases.^25^ Utilities are an alternative to disability weights and are recommended for cost-effectiveness analyses for HICs.^8,30^ Utilities are intended to be context-specific, reported from the patient or societal perspective, and can account for health equity.^30,^^31^ However, utilities in LMICs are scarce compared to those in HICs. This is especially the case for surgical diseases relative to pharmaceutical interventions and the lack of context-appropriate utilities for these populations can further disadvantage them in LMIC resource allocation decisions. Thus, opportunities exist for further investigations that consider utilities, costs, complications, and multidisciplinary management for CL/P from LMIC participants to provide the context-relevant data that is currently missing. Collaborations to provide this evidence can help inform decisions that can lead to a sustainable, multidisciplinary, and longitudinal standard of surgical care that is acceptable in HICs.

## Conclusions

Existing economic evaluations suggest primary CL/P repair is cost-effective, however, there is a need for context-specific cost and utility data for primary CL/P repair, complications, and existing multidisciplinary management in LMICs. Context-specific local data will make future CEAs more relevant to local decision-makers and lead to better-informed resource allocation decisions in LMICs.

## Supplemental Material

sj-docx-1-cpc-10.1177_10556656221111028 - Supplemental material for A Systematic Review of the Cost-Effectiveness of Cleft Care in Low- and Middle-Income Countries: What is Needed? Supplemental material, sj-docx-1-cpc-10.1177_10556656221111028 for A Systematic Review of the Cost-Effectiveness of Cleft Care in Low- and Middle-Income Countries: What is Needed? by Karen Y. Chung, George Ho, Aysegul Erman, Joanna M. Bielecki, Christopher R. Forrest and Beate Sander in The Cleft Palate Craniofacial Journal
