# Supplementary material for: A Systematic Review of the Cost-Effectiveness of Cleft Care in Low- and Middle-Income Countries: What is Needed?
Source: Cleft Palate Craniofac J. 2022 Jul 3;60(12):1600–8. doi: 10.1177/10556656221111028 (PMC10588273; doi:10.1177/10556656221111028)

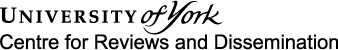


Systematic review

# * Review title.

Give the title of the review in English

The cost-effectiveness of cleft lip and/or palate care in low- and middle- income countries: a systematic review

# Original language title.

For reviews in languages other than English, give the title in the original language. This will be displayed with the English language title.

# * Anticipated or actual start date.

Give the date the systematic review started or is expected to start. 27/08/2019

# * Anticipated completion date.

Give the date by which the review is expected to be completed.

10/25/2021

# * Stage of review at time of this submission.

Tick the boxes to show which review tasks have been started and which have been completed. Update this field each time any amendments are made to a published record.

**Reviews that have started data extraction (at the time of initial submission) are not eligible for inclusion in PROSPERO**. If there is later evidence that incorrect status and/or completion date has been supplied, the published PROSPERO record will be marked as retracted.

This field uses answers to initial screening questions. It cannot be edited until after registration. The review has not yet started: No

**Review stage Started Completed**

Preliminary searches Yes Yes

Piloting of the study selection process Yes Yes

Formal screening of search results against eligibility criteria Yes Yes

Data extraction Yes Yes

Risk of bias (quality) assessment Yes Yes

Data analysis Yes Yes

Provide any other relevant information about the stage of the review here.

# * Named contact.

The named contact is the guarantor for the accuracy of the information in the register record. This may be any member of the review team.

Karen Chung

# Email salutation (e.g. "Dr Smith" or "Joanne") for correspondence:

Dr Chung

# * Named contact email.

Give the electronic email address of the named contact. [ky.chung@mail.utoronto.ca](mailto:ky.chung@mail.utoronto.ca)

# Named contact address

Give the full institutional/organisational postal address for the named contact.

University of Toronto

# Named contact phone number.

Give the telephone number for the named contact, including international dialling code. 9057197919

# * Organisational affiliation of the review.

Full title of the organisational affiliations for this review and website address if available. This field may be completed as 'None' if the review is not affiliated to any organisation.

University of Toronto

# Organisation web address:

1. * Review team members and their organisational affiliations.

Give the personal details and the organisational affiliations of each member of the review team. Affiliation refers to groups or organisations to which review team members belong. **NOTE: email and country now MUST be entered for each person, unless you are amending a published record.**

Dr Karen Chung. University of Toronto Mr George Ho. University of Toronto

Miss Joanna M. Bielecki. Toronto Health Economics and Technology Assessment Dr Christopher Forrest. University of Toronto

Dr Beate Sander. University Health Network, Toronto

# * Funding sources/sponsors.

Details of the individuals, organizations, groups, companies or other legal entities who have funded or sponsored the review.

Not applicable

# Grant number(s)

State the funder, grant or award number and the date of award

# * Conflicts of interest.

List actual or perceived conflicts of interest (financial or academic). None

# Collaborators.

Give the name and affiliation of any individuals or organisations who are working on the review but who are not listed as review team members. **NOTE: email and country must be completed for each person, unless you are amending a published record.**

# * Review question.

State the review question(s) clearly and precisely. It may be appropriate to break very broad questions down into a series of related more specific questions. Questions may be framed or refined using PI(E)COS or similar where relevant.

What is the cost-effectiveness of cleft lip and/or palate management in low- and middle- income countries (as defined by the World Bank)?

# * Searches.

State the sources that will be searched (e.g. Medline). Give the search dates, and any restrictions (e.g. language or publication date). Do NOT enter the full search strategy (it may be provided as a link or attachment below.)

We will conduct this systematic review in accordance with PRISMA (Preferred Reporting Items for Systematic Reviews and Meta-Analyses) guidelines.

The search strategy will be designed and executed by an Information Specialist (JB) with expertise in systematic review in eleven electronic databases: Global Health Cost Effectiveness Analysis (GH CEA) Registry, Cost- Effectiveness Analysis (CEA) Registry, Ovid MEDLINE, Ovid EMBASE, Global Index Medicus, ScHARRHUD database, Cochrane Database of Systematic Reviews database, and the Center for Reviews and Dissemination (CRD) Databases, which includes the Health Technology Assessment Database (HTA), HTA Database Canadian Repository, the NHS Economic Evaluation Database (NHS EED) and the

Database of Abstracts of Reviews of Effects (DARE). Bibliographic records were published on DARE and NHS EED until 31st March 2015.

Literature search strategies will be designed using key words and medical subject headings (MeSH) and text words related to the following concepts: cleft lip, cleft palate, cost-effectiveness and economic evaluations.

The search strategy will be initially developed in MEDLINE, and then adapted to the syntax and subject headings of the other databases. PROSPERO will be searched for ongoing or recently completed systematic reviews.

Studies published from 2000 to the present (December 29 2020)

Additionally, grey literature and bibliographies of identified publications will be hand searched for potentially relevant articles.The search will be updated toward the end of the review.

Review process: two reviewers (KC, GH) will assess the titles, abstracts, and full texts for inclusion using DistillerSR. Disagreements will be resolved by third party experts (CF, BS)

# URL to search strategy.

Upload a file with your search strategy, or an example of a search strategy for a specific database, (including the keywords) in pdf or word format. In doing so you are consenting to the file being made publicly accessible. Or provide a URL or link to the strategy. Do NOT provide links to your search **results**.

[https://www.crd.york.ac.uk/PROSPEROFILES/148402_STRATEGY_20211026.pdf](http://www.crd.york.ac.uk/PROSPEROFILES/148402_STRATEGY_20211026.pdf)

Alternatively, upload your search strategy to CRD in pdf format. Please note that by doing so you are consenting to the file being made publicly accessible.

Do not make this file publicly available until the review is complete

# * Condition or domain being studied.

Give a short description of the disease, condition or healthcare domain being studied in your systematic review.

Cleft lip and/or palate (CLP) is the most common craniofacial congenital anomaly worldwide. Untreated CLP is associated with poor feeding, poor oral health, poor speech, and ear infections which is exacerbated with delays or barriers to care in low-resource settings. There are still areas in low-middle income countries, where CLP education and awareness of the disease is limited, and children with CLP may not be seen as human. Children with CLP can experience extreme social rejection, with negative downstream sequelae.

They can be refused admission to school on the basis that it would frighten other children, or may refuse to go to school because of taunting. Yet, the healthcare resources in low-middle income countries are scarce and CLP is not a priority.

# * Participants/population.

Specify the participants or populations being studied in the review. The preferred format includes details of both inclusion and exclusion criteria.

All cleft lip and/or palate patients, including revisions, non-syndromic and syndromic patients within low- middle income countries (as defined by the World Bank), or within low-resource settings independent of country.

# * Intervention(s), exposure(s).

Give full and clear descriptions or definitions of the interventions or the exposures to be reviewed. The preferred format includes details of both inclusion and exclusion criteria.

Cleft lip and/or palate interventions

# * Comparator(s)/control.

Where relevant, give details of the alternatives against which the intervention/exposure will be compared (e.g. another intervention or a non-exposed control group). The preferred format includes details of both inclusion and exclusion criteria.

No cleft lip and/or palate therapeutic or surgical procedures

# * Types of study to be included.

Give details of the study designs (e.g. RCT) that are eligible for inclusion in the review. The preferred format includes both inclusion and exclusion criteria. If there are no restrictions on the types of study, this should be stated.

Full economic evaluations, e.g., cost-benefit analyses, cost-effectiveness analyses and cost-utility analyses Inclusion criteria:

1. Full economic evaluations (e.g. cost-benefit analysis, cost-effectiveness analysis and cost-utility analysis).
2. Studies published from 2000 to December 29 2020
3. Studies conducted in low- and middle-income countries as defined by the World Bank in 2020 (if available)
4. Studies conducted in all rural or low-resource settings, independent of country
5. English language studies due to feasibility and resource constraints.
6. Specific to cleft lip and/or palate

Exclusion criteria:

1. Studies that are not conducted in from low- and middle-income countries or not conducted in rural or low- resource settings.
2. Studies not in English
3. Other forms of economic evaluations (e.g. cost-minimization, cost-consequence and cost-of-illness studies).
4. Not relevant to cleft lip and/or palate

# Context.

Give summary details of the setting or other relevant characteristics, which help define the inclusion or exclusion criteria.

# * Main outcome(s).

Give the pre-specified main (most important) outcomes of the review, including details of how the outcome is defined and measured and when these measurement are made, if these are part of the review inclusion criteria.

The cost-effectiveness of the intervention, assessed using incremental ratios of costs per unit of benefit (e.g.

cost per quality adjusted life year, cost per cases averted, cost per life year gained, cost-benefit ratio).

# Measures of effect

Please specify the effect measure(s) for you main outcome(s) e.g. relative risks, odds ratios, risk difference, and/or 'number needed to treat.

None

# * Additional outcome(s).

List the pre-specified additional outcomes of the review, with a similar level of detail to that required for main outcomes. Where there are no additional outcomes please state ‘None’ or ‘Not applicable’ as appropriate

to the review

Hospital Length of stay, peri-operative mortality, complications

# Measures of effect

Please specify the effect measure(s) for you additional outcome(s) e.g. relative risks, odds ratios, risk difference, and/or 'number needed to treat.

None

# * Data extraction (selection and coding).

Describe how studies will be selected for inclusion. State what data will be extracted or obtained. State how this will be done and recorded.

Two reviewers (KC, GH) will complete title and abstract, and full-text screening independently. Conflicts will be discussed and if not resolved through initial discussion, then through discussion with a third reviewer. Two reviewers will extract and populate data extraction tables for the variables below, guided by CHEERS (Consolidated Health Economics Evaluation and Reporting Statement). We will attempt to contact authors for clarification or missing data, and any discrepancies in data extraction will be resolved through discussion with a third reviewer.

Study characteristics:

- Study author and year;
- Country of study;
- Study design and type of model;
- Key assumptions;
- Study perspective;
- Time horizon;
- Discounting;
- Intervention and comparison:
- Choice of health outcomes (measurement of effectiveness);
- Valuation of preference-based outcomes;
- Valuation of costs (currency, year);
- Use of cost-effectiveness threshold for conclusions;
- Funding source of the studies; Patient/population characteristics:
- Target population;
- Age range;
- Setting/location;
- Health conditions;

Study results:

- Base-case results (primary and secondary outcomes of interest);
- Sensitivity analysis results.

# * Risk of bias (quality) assessment.

State which characteristics of the studies will be assessed and/or any formal risk of bias/quality assessment tools that will be used.

Two reviewers (KC, GH) will assess the quality of eligible economic evaluations through critical appraisals specific to study designs, and will resolve disagreements through discussion.

Studies will be assessed using the Joanna Briggs Institute (JBI) Critical Appraisal Checklist for Economic Evaluations.

# * Strategy for data synthesis.

Describe the methods you plan to use to synthesise data. This **must not be generic text** but should be **specific to your review** and describe how the proposed approach will be applied to your data. If meta- analysis is planned, describe the models to be used, methods to explore statistical heterogeneity, and software package to be used.

We will conduct a descriptive analysis of the study characteristics, and will summarize and report cost- effectiveness outcomes unadjusted, and adjusted to 2020, in USD, using purchasing power parities. We will also summarize and report the secondary health outcomes of interest (complications, perioperative mortality and hospital length of stay if reported), and analysis may be stratified by age, disease status (cleft lip and/or cleft palate), study type, as well as by regions (i.e. continents). We will also report the range of cost- effectiveness based on expressed units, and will plot them on a graph (e.g. scatterplot, or histogram).

# * Analysis of subgroups or subsets.

State any planned investigation of ‘subgroups’. Be clear and specific about which type of study or participant will be included in each group or covariate investigated. State the planned analytic approach.

Further exploration will be by country, age, disease status (cleft lip, cleft lip and/or palate and cleft palate) and study type

# * Type and method of review.

Select the type of review, review method and health area from the lists below.

Type of review Cost effectiveness Yes

Diagnostic No

Epidemiologic No

Individual patient data (IPD) meta-analysis No

Intervention No

Living systematic review No

Meta-analysis No

Methodology No

Narrative synthesis No

Network meta-analysis No

Pre-clinical No

Prevention No

Prognostic No

Prospective meta-analysis (PMA) No

Review of reviews No

Service delivery No

Synthesis of qualitative studies No

Systematic review Yes

Other No

Health area of the review Alcohol/substance misuse/abuse No

Blood and immune system No

Cancer No

Cardiovascular No

Care of the elderly No

Child health Yes

Complementary therapies No

COVID-19

No

Crime and justice No

Dental No

Digestive system No

Ear, nose and throat No

Education No

Endocrine and metabolic disorders No

Eye disorders No

General interest No

Genetics No

Health inequalities/health equity No

Infections and infestations No

International development

No

Mental health and behavioural conditions No

Musculoskeletal No

Neurological No

Nursing No

Obstetrics and gynaecology No

Oral health Yes

Palliative care No

Perioperative care No

Physiotherapy No

Pregnancy and childbirth No

Public health (including social determinants of health) No

Rehabilitation No

Respiratory disorders No

Service delivery No

Skin disorders No

Social care No

Surgery Yes

Tropical Medicine No

Urological No

Wounds, injuries and accidents No

Violence and abuse No

# Language.

Select each language individually to add it to the list below, use the bin icon to remove any added in error. English

There is not an English language summary

# * Country.

Select the country in which the review is being carried out. For multi-national collaborations select all the countries involved.

Canada

# Other registration details.

Name any other organisation where the systematic review title or protocol is registered (e.g. Campbell, or The Joanna Briggs Institute) together with any unique identification number assigned by them. If extracted data will be stored and made available through a repository such as the Systematic Review Data Repository (SRDR), details and a link should be included here. If none, leave blank.

# Reference and/or URL for published protocol.

If the protocol for this review is published provide details (authors, title and journal details, preferably in Vancouver format)

Add web link to the published protocol.

Or, upload your published protocol here in pdf format. Note that the upload will be publicly accessible. No I do not make this file publicly available until the review is complete

Please note that the information required in the PROSPERO registration form must be completed in full even if access to a protocol is given.

# Dissemination plans.

Do you intend to publish the review on completion?

Yes

Give brief details of plans for communicating review findings.? Local, national and international platforms

# Keywords.

Give words or phrases that best describe the review. Separate keywords with a semicolon or new line. Keywords help PROSPERO users find your review (keywords do not appear in the public record but are included in searches). Be as specific and precise as possible. Avoid acronyms and abbreviations unless these are in wide use.

Economic Evaluation; Child Health; Cleft lip and/or Palate; Global Health; Cost-Effectiveness

# Details of any existing review of the same topic by the same authors.

If you are registering an update of an existing review give details of the earlier versions and include a full bibliographic reference, if available.

# * Current review status.

Update review status when the review is completed and when it is published.New registrations must be ongoing so this field is not editable for initial submission.

Please provide anticipated publication date Review_Completed_not_published

# Any additional information.

Provide any other information relevant to the registration of this review.

# Details of final report/publication(s) or preprints if available.

Leave empty until publication details are available OR you have a link to a preprint (NOTE: this field is not editable for initial submission). List authors, title and journal details preferably in Vancouver format.

Give the link to the published review or preprint.

### Topic: The Cost-Effectiveness of Cleft Lip and/or Palate Surgery in Global Health: A Systematic Review

**Search done for:** Karen Chung (kchung@qmed.ca)

**Performed by:** George Ho

**Original search date:** Jan 21, 2019

**Update search Date:** Dec 29, 2020

### Database(s) to be searched:

- Ovid Medline (R) and Medline (R) In-Process and Other Non-Indexed Citations
- Ovid Embase
- Cochrane Database of Systematic Reviews
- Global Index Medicus
- Centre for Reviews and Dissemination (CRD) Databases:
  - Health Technology Assessment Database (HTA)
  - NHS Economic Evaluation Database (NHS EED)
  - Database of Abstracts of Reviews of Effects (DARE)
  - HTA Database Canadian Repository - **no longer available**
- ScHARRHUD database
- Global Health Cost Effectiveness Analysis (GH CEA) Registry
- Cost- Effectiveness Analysis (CEA) Registry

**Limits:** 2000 to the present; English language

**MEDLINE SEARCH - 495 articles Date:** Dec 29, 2020

**Databases searched:** Ovid MEDLINE: Epub Ahead of Print, In-Process & Other Non-Indexed Citations, Ovid MEDLINE® Daily and Ovid MEDLINE® 1946-Present

### Search Strategy:


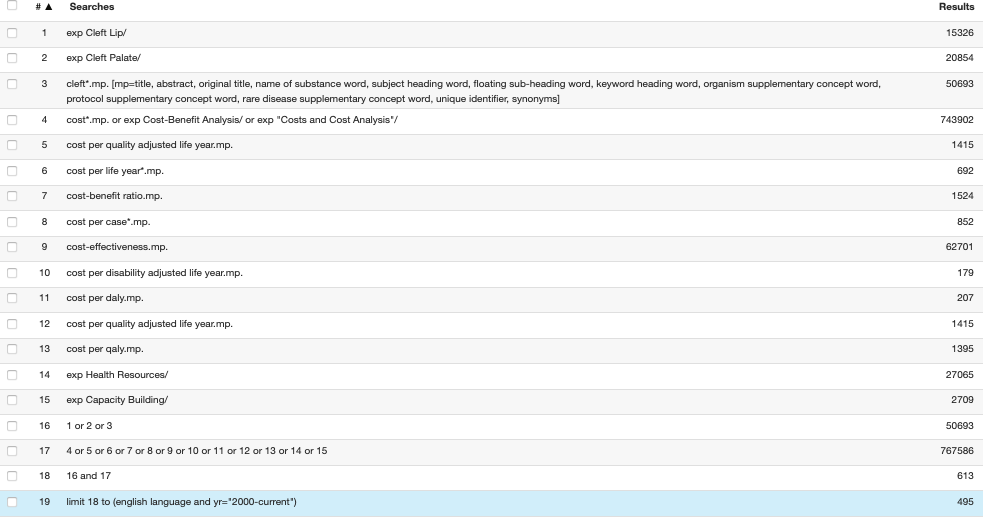


**EMBASE SEARCH - 879 articles Date:** Dec 29, 2020

**Databases searched:** Embase Classic+Embase 1947 to 2020 December 28

### Search Strategy:


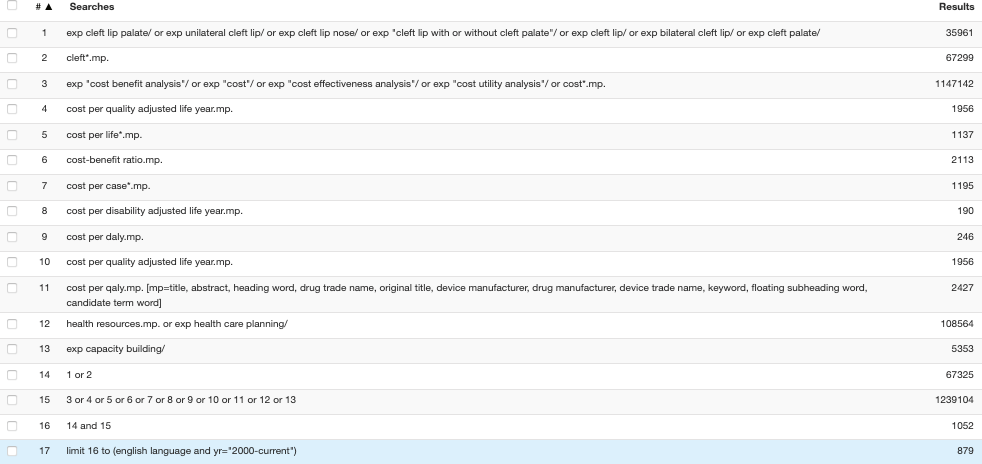


**Cochrane Database of Systematic Reviews - 2 articles Databases searched:** Cochrane Reviews and Cochrane Protocols **Date:** Dec 28, 2020

###
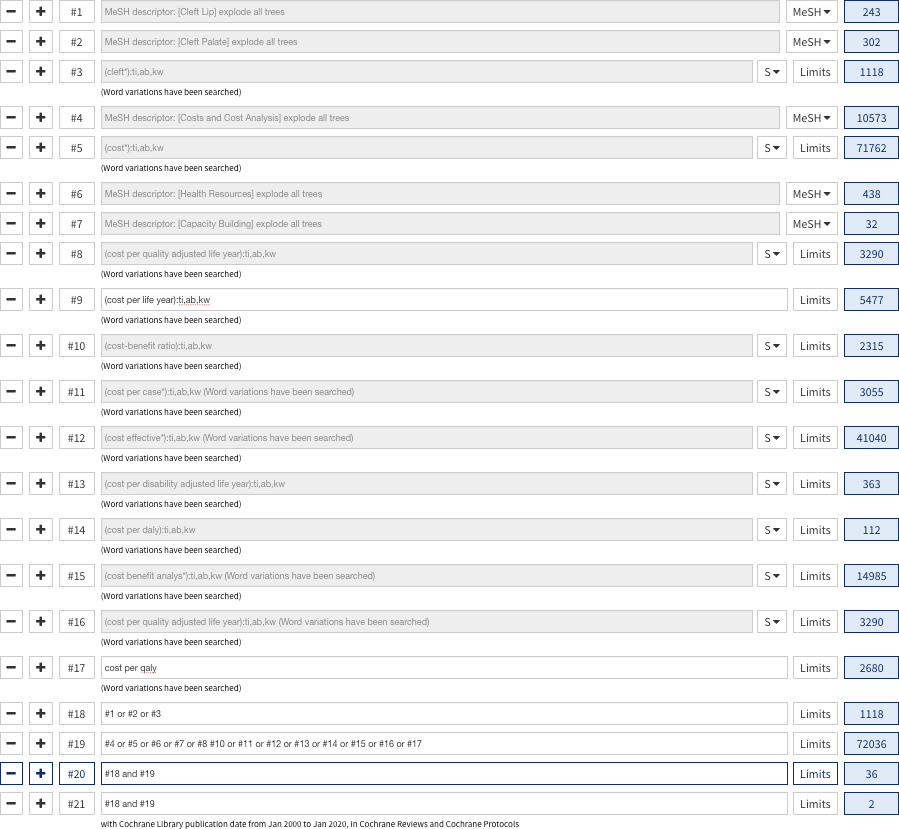
Search Strategy:

**Global Index Medicus - 1471 Date:** Dec 29, 2020

**Databases searched:** Global Index Medicus – GIM; Regional Indexes: AIM (Africa), LILACS (Americans), IMEMR (Eastern Mediterranean), IMSEAR (South-East Asia), WPRIM (Western Pacific)

### Search Strategy:

| **Searches** | **Results** |
| --- | --- |
| tw:((tw:(cleft lip)) OR (tw:(cleft palate)) OR (tw:(cleft*)) AND (tw:(cost*)) OR (tw:(cost benefit analysis)) OR (tw:(cost analysis)) OR (tw:(cost per quality adjusted life year)) OR (tw:(cost per life year gained)) OR (tw:(cost-benefit ratio)) OR (tw:(cost per cases averted)) OR (tw:(cost-effectiveness)) OR (tw:(cost per disability adjusted life year)) OR (tw:(cost per daly)) OR (tw:(health resource*)) OR  (tw:(capacity building))) AND ( la:("en")) AND (year_cluster:[2000 TO 2020]) | 1471 |

[https://pesquisa.bvsalud.org/gim/?output=site&lang=en&from=1&sort=&format=summary&count=20&fb=&page=1&filter%5Bla%5D%5B%5D=en&range](https://pesquisa.bvsalud.org/gim/?output=site&lang=en&from=1&sort&format=summary&count=20&fb&page=1&filter%5Bla%5D%5B%5D=en&range_year_start=2000&range_year_end=2020&index=tw&q=tw%3A%28%28tw%3A%28cleft%2Blip%29%29%2BOR%2B%28tw%3A%28cleft%2Bpalate%29%29%2BOR%2B%28tw%3A%28cleft%2A%29%29%2BAND%2B%28tw%3A%28cost%2A%29%29%2BOR%2B%28tw%3A%28cost%2Bbenefit%2Banalysis%29%29%2BOR%2B%28tw%3A%28cost%2Banalysis%29%29%2BOR%2B%28tw%3A%28cost%2Bper%2Bquality%2Badjusted%2Blife%2Byear%29%29%2BOR%2B%28tw%3A%28cost%2Bper%2Blife%2Byear%2Bgained%29%29%2BOR%2B%28tw%3A%28cost-benefit%2Bratio%29%29%2BOR%2B%28tw%3A%28cost%2Bper%2Bcases%2Baverted%29%29%2BOR%2B%28tw%3A%28cost-effectiveness%29%29%2BOR%2B%28tw%3A%28cost%2Bper%2Bdisability%2Badjusted%2Blife%2Byear%29%29%2BOR%2B%28tw%3A%28cost%2Bper%2Bdaly%29%29%2BOR%2B%28tw%3A%28health%2Bresource%2A%29%29%2BOR%2B%28tw%3A%28capacity%2Bbuilding%29%29%29&search_form_submit)

[_year_start=2000&range_year_end=2020&index=tw&q=tw%3A%28%28tw%3A%28cleft+lip%29%29+OR+%28tw%3A%28cleft+palate%29%29+OR+%28tw](https://pesquisa.bvsalud.org/gim/?output=site&lang=en&from=1&sort&format=summary&count=20&fb&page=1&filter%5Bla%5D%5B%5D=en&range_year_start=2000&range_year_end=2020&index=tw&q=tw%3A%28%28tw%3A%28cleft%2Blip%29%29%2BOR%2B%28tw%3A%28cleft%2Bpalate%29%29%2BOR%2B%28tw%3A%28cleft%2A%29%29%2BAND%2B%28tw%3A%28cost%2A%29%29%2BOR%2B%28tw%3A%28cost%2Bbenefit%2Banalysis%29%29%2BOR%2B%28tw%3A%28cost%2Banalysis%29%29%2BOR%2B%28tw%3A%28cost%2Bper%2Bquality%2Badjusted%2Blife%2Byear%29%29%2BOR%2B%28tw%3A%28cost%2Bper%2Blife%2Byear%2Bgained%29%29%2BOR%2B%28tw%3A%28cost-benefit%2Bratio%29%29%2BOR%2B%28tw%3A%28cost%2Bper%2Bcases%2Baverted%29%29%2BOR%2B%28tw%3A%28cost-effectiveness%29%29%2BOR%2B%28tw%3A%28cost%2Bper%2Bdisability%2Badjusted%2Blife%2Byear%29%29%2BOR%2B%28tw%3A%28cost%2Bper%2Bdaly%29%29%2BOR%2B%28tw%3A%28health%2Bresource%2A%29%29%2BOR%2B%28tw%3A%28capacity%2Bbuilding%29%29%29&search_form_submit)

[%3A%28cleft*%29%29+AND+%28tw%3A%28cost*%29%29+OR+%28tw%3A%28cost+benefit+analysis%29%29+OR+%28tw%3A%28cost+analysis%29%29+](https://pesquisa.bvsalud.org/gim/?output=site&lang=en&from=1&sort&format=summary&count=20&fb&page=1&filter%5Bla%5D%5B%5D=en&range_year_start=2000&range_year_end=2020&index=tw&q=tw%3A%28%28tw%3A%28cleft%2Blip%29%29%2BOR%2B%28tw%3A%28cleft%2Bpalate%29%29%2BOR%2B%28tw%3A%28cleft%2A%29%29%2BAND%2B%28tw%3A%28cost%2A%29%29%2BOR%2B%28tw%3A%28cost%2Bbenefit%2Banalysis%29%29%2BOR%2B%28tw%3A%28cost%2Banalysis%29%29%2BOR%2B%28tw%3A%28cost%2Bper%2Bquality%2Badjusted%2Blife%2Byear%29%29%2BOR%2B%28tw%3A%28cost%2Bper%2Blife%2Byear%2Bgained%29%29%2BOR%2B%28tw%3A%28cost-benefit%2Bratio%29%29%2BOR%2B%28tw%3A%28cost%2Bper%2Bcases%2Baverted%29%29%2BOR%2B%28tw%3A%28cost-effectiveness%29%29%2BOR%2B%28tw%3A%28cost%2Bper%2Bdisability%2Badjusted%2Blife%2Byear%29%29%2BOR%2B%28tw%3A%28cost%2Bper%2Bdaly%29%29%2BOR%2B%28tw%3A%28health%2Bresource%2A%29%29%2BOR%2B%28tw%3A%28capacity%2Bbuilding%29%29%29&search_form_submit) [OR+%28tw%3A%28cost+per+quality+adjusted+life+year%29%29+OR+%28tw%3A%28cost+per+life+year+gained%29%29+OR+%28tw%3A%28cost-](https://pesquisa.bvsalud.org/gim/?output=site&lang=en&from=1&sort&format=summary&count=20&fb&page=1&filter%5Bla%5D%5B%5D=en&range_year_start=2000&range_year_end=2020&index=tw&q=tw%3A%28%28tw%3A%28cleft%2Blip%29%29%2BOR%2B%28tw%3A%28cleft%2Bpalate%29%29%2BOR%2B%28tw%3A%28cleft%2A%29%29%2BAND%2B%28tw%3A%28cost%2A%29%29%2BOR%2B%28tw%3A%28cost%2Bbenefit%2Banalysis%29%29%2BOR%2B%28tw%3A%28cost%2Banalysis%29%29%2BOR%2B%28tw%3A%28cost%2Bper%2Bquality%2Badjusted%2Blife%2Byear%29%29%2BOR%2B%28tw%3A%28cost%2Bper%2Blife%2Byear%2Bgained%29%29%2BOR%2B%28tw%3A%28cost-benefit%2Bratio%29%29%2BOR%2B%28tw%3A%28cost%2Bper%2Bcases%2Baverted%29%29%2BOR%2B%28tw%3A%28cost-effectiveness%29%29%2BOR%2B%28tw%3A%28cost%2Bper%2Bdisability%2Badjusted%2Blife%2Byear%29%29%2BOR%2B%28tw%3A%28cost%2Bper%2Bdaly%29%29%2BOR%2B%28tw%3A%28health%2Bresource%2A%29%29%2BOR%2B%28tw%3A%28capacity%2Bbuilding%29%29%29&search_form_submit) [benefit+ratio%29%29+OR+%28tw%3A%28cost+per+cases+averted%29%29+OR+%28tw%3A%28cost-](https://pesquisa.bvsalud.org/gim/?output=site&lang=en&from=1&sort&format=summary&count=20&fb&page=1&filter%5Bla%5D%5B%5D=en&range_year_start=2000&range_year_end=2020&index=tw&q=tw%3A%28%28tw%3A%28cleft%2Blip%29%29%2BOR%2B%28tw%3A%28cleft%2Bpalate%29%29%2BOR%2B%28tw%3A%28cleft%2A%29%29%2BAND%2B%28tw%3A%28cost%2A%29%29%2BOR%2B%28tw%3A%28cost%2Bbenefit%2Banalysis%29%29%2BOR%2B%28tw%3A%28cost%2Banalysis%29%29%2BOR%2B%28tw%3A%28cost%2Bper%2Bquality%2Badjusted%2Blife%2Byear%29%29%2BOR%2B%28tw%3A%28cost%2Bper%2Blife%2Byear%2Bgained%29%29%2BOR%2B%28tw%3A%28cost-benefit%2Bratio%29%29%2BOR%2B%28tw%3A%28cost%2Bper%2Bcases%2Baverted%29%29%2BOR%2B%28tw%3A%28cost-effectiveness%29%29%2BOR%2B%28tw%3A%28cost%2Bper%2Bdisability%2Badjusted%2Blife%2Byear%29%29%2BOR%2B%28tw%3A%28cost%2Bper%2Bdaly%29%29%2BOR%2B%28tw%3A%28health%2Bresource%2A%29%29%2BOR%2B%28tw%3A%28capacity%2Bbuilding%29%29%29&search_form_submit) [effectiveness%29%29+OR+%28tw%3A%28cost+per+disability+adjusted+life+year%29%29+OR+%28tw%3A%28cost+per+daly%29%29+OR+%28tw%3A%28](https://pesquisa.bvsalud.org/gim/?output=site&lang=en&from=1&sort&format=summary&count=20&fb&page=1&filter%5Bla%5D%5B%5D=en&range_year_start=2000&range_year_end=2020&index=tw&q=tw%3A%28%28tw%3A%28cleft%2Blip%29%29%2BOR%2B%28tw%3A%28cleft%2Bpalate%29%29%2BOR%2B%28tw%3A%28cleft%2A%29%29%2BAND%2B%28tw%3A%28cost%2A%29%29%2BOR%2B%28tw%3A%28cost%2Bbenefit%2Banalysis%29%29%2BOR%2B%28tw%3A%28cost%2Banalysis%29%29%2BOR%2B%28tw%3A%28cost%2Bper%2Bquality%2Badjusted%2Blife%2Byear%29%29%2BOR%2B%28tw%3A%28cost%2Bper%2Blife%2Byear%2Bgained%29%29%2BOR%2B%28tw%3A%28cost-benefit%2Bratio%29%29%2BOR%2B%28tw%3A%28cost%2Bper%2Bcases%2Baverted%29%29%2BOR%2B%28tw%3A%28cost-effectiveness%29%29%2BOR%2B%28tw%3A%28cost%2Bper%2Bdisability%2Badjusted%2Blife%2Byear%29%29%2BOR%2B%28tw%3A%28cost%2Bper%2Bdaly%29%29%2BOR%2B%28tw%3A%28health%2Bresource%2A%29%29%2BOR%2B%28tw%3A%28capacity%2Bbuilding%29%29%29&search_form_submit) [health+resource*%29%29+OR+%28tw%3A%28capacity+building%29%29%29&search_form_submit=](https://pesquisa.bvsalud.org/gim/?output=site&lang=en&from=1&sort&format=summary&count=20&fb&page=1&filter%5Bla%5D%5B%5D=en&range_year_start=2000&range_year_end=2020&index=tw&q=tw%3A%28%28tw%3A%28cleft%2Blip%29%29%2BOR%2B%28tw%3A%28cleft%2Bpalate%29%29%2BOR%2B%28tw%3A%28cleft%2A%29%29%2BAND%2B%28tw%3A%28cost%2A%29%29%2BOR%2B%28tw%3A%28cost%2Bbenefit%2Banalysis%29%29%2BOR%2B%28tw%3A%28cost%2Banalysis%29%29%2BOR%2B%28tw%3A%28cost%2Bper%2Bquality%2Badjusted%2Blife%2Byear%29%29%2BOR%2B%28tw%3A%28cost%2Bper%2Blife%2Byear%2Bgained%29%29%2BOR%2B%28tw%3A%28cost-benefit%2Bratio%29%29%2BOR%2B%28tw%3A%28cost%2Bper%2Bcases%2Baverted%29%29%2BOR%2B%28tw%3A%28cost-effectiveness%29%29%2BOR%2B%28tw%3A%28cost%2Bper%2Bdisability%2Badjusted%2Blife%2Byear%29%29%2BOR%2B%28tw%3A%28cost%2Bper%2Bdaly%29%29%2BOR%2B%28tw%3A%28health%2Bresource%2A%29%29%2BOR%2B%28tw%3A%28capacity%2Bbuilding%29%29%29&search_form_submit)

**CRD Databases SEARCH - 14 articles Date:** Dec 29, 2020

### Databases searched:

Centre for Reviews and Dissemination (CRD) Databases: Health Technology Assessment Database (HTA), NHS Economic Evaluation Database (NHS EED), Database of Abstracts of Reviews of Effects (DARE)

**Search Strategy:**


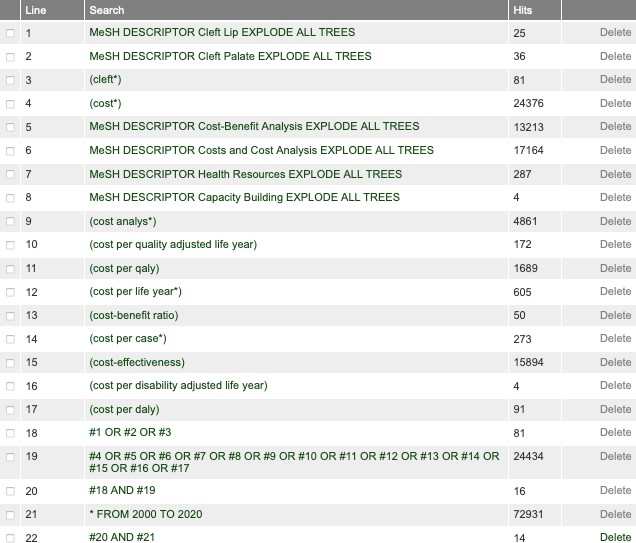


**HTA Database Canadian Repository - DATABASE NO LONGER AVAILABLE**

**ScHARRHUD database - 0 articles**

**Date:** Dec 29, 2020

**Databases searched:** ScHARRHUD database

**Search Strategy:** 0 results for cleft* OR cleft palate OR cleft lip (multiple variations searched)


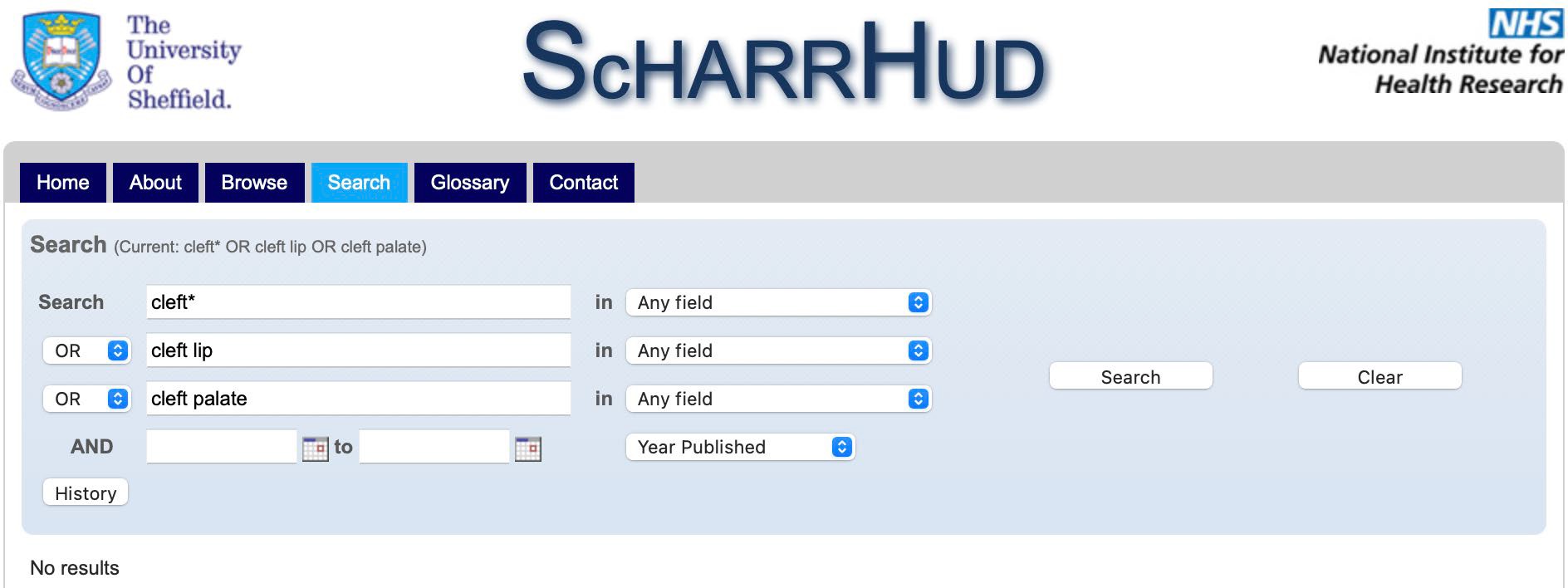

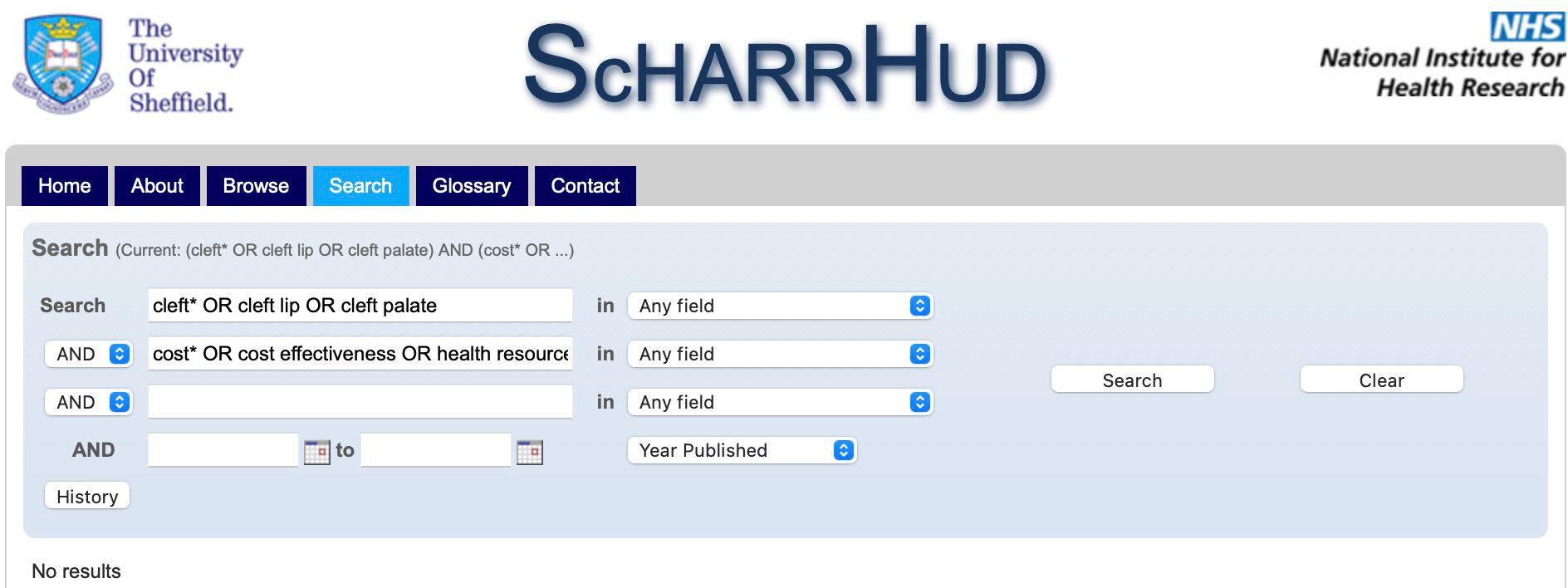


**CEA & GH CEA Registry Date:** Dec 29, 2020 **Databases searched:**

Global Health Cost Effectiveness Analysis (GH CEA) Registry - **1 article (duplicate from pubmed)**

Cost- Effectiveness Analysis (CEA) Registry - **20 articles Global Health Cost Effectiveness Analysis (GH CEA) Registry**


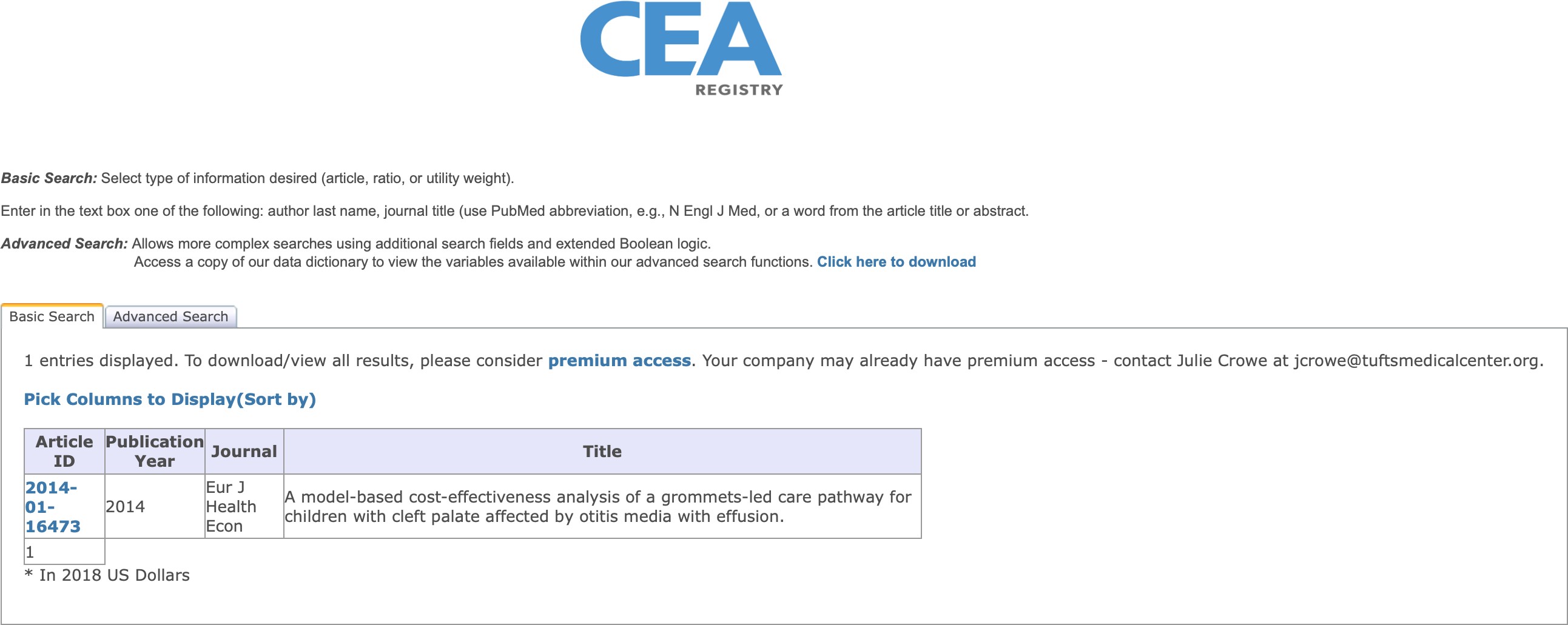


###
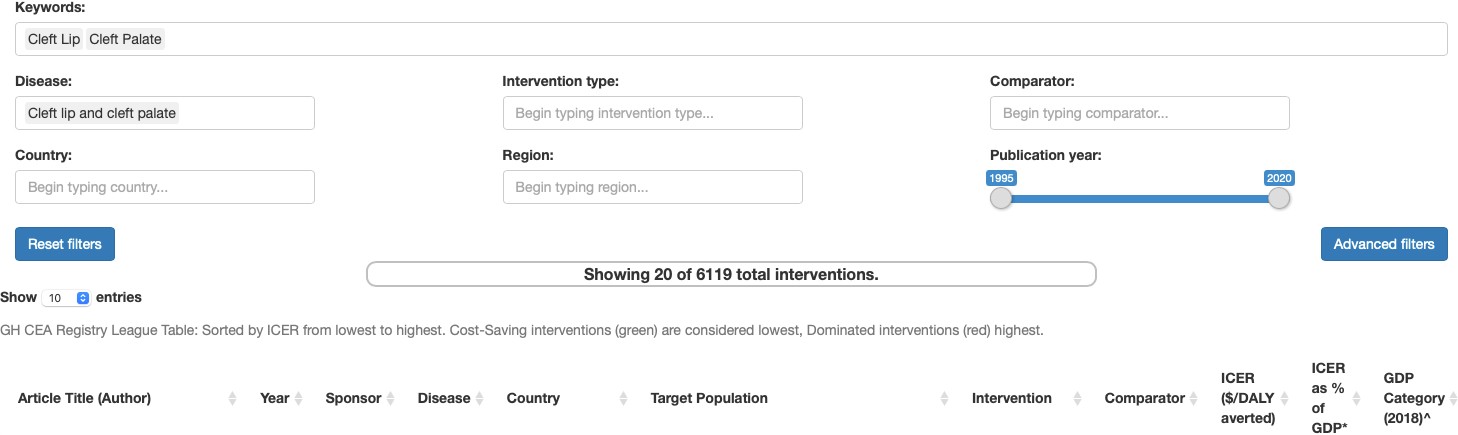
Cost- Effectiveness Analysis (CEA) Registry

**Topic: The Cost-Effectiveness of Cleft Lip and/or Palate Surgery in Global Health: A Systematic Review**

**Search done for:** Karen Chung (kchung@qmed.ca)

**Performed by:** Joanna Bielecki ([joanna.bielecki@theta.utoronto.ca](mailto:joanna.bielecki@theta.utoronto.ca))

**Original search date:** Jan 21, 2019

**Update search Date:** Feb 12, 2020

### Database(s) to be searched:

Ovid Medline (R) and Medline (R) In-Process and Other Non-Indexed Citations Ovid Embase

Cochrane Database of Systematic Reviews Global Index Medicus

Centre for Reviews and Dissemination (CRD) Databases: Health Technology Assessment Database (HTA) NHS Economic Evaluation Database (NHS EED) Database of Abstracts of Reviews of Effects (DARE) HTA Database Canadian Repository

ScHARRHUD database

Global Health Cost Effectiveness Analysis (GH CEA) Registry Cost- Effectiveness Analysis (CEA) Registry

Limits: 2000 to the present; English language

## MEDLINE SEARCH

**Date:** Jan 21, 2019

**Search Strategy name:** "CLP and EconEval - MEDLINE - FINAL"

### Databases searched:

Ovid MEDLINE: Epub Ahead of Print, In-Process & Other Non-Indexed Citations, Ovid MEDLINE® Daily and Ovid MEDLINE® 1946-Present

### Search Strategy:

| **#** | **Searches** | **Results** |
| --- | --- | --- |
| 1 | Cleft Palate/ or Cleft Lip/ or ((Cleft adj3 (Palate? or lip? or Orofacial)) or (palate adj3 surg$) or Harelip?).ti,ab,kf. | 28119 |
| 2 | exp "costs and cost analysis"/ or costs.tw. or cost effective$.tw. | 409146 |
| 3 | (Cost$ or cost benefit analys$ or health care costs).mp. | 631666 |
| 4 | 2 or 3 | 642203 |
| 5 | 1 and 4 | 338 |
| 6 | limit 5 to (english language and yr="2000 -Current") | 261 |

**Update Date:** Feb 12, 2020

**Search Strategy name:** "CLP and EconEval - MEDLINE - FINAL"

### Database(s): Ovid MEDLINE: Epub Ahead of Print, In-Process & Other Non-Indexed Citations, Ovid MEDLINE® Daily and Ovid MEDLINE® 1946-Present

Search Strategy:

| **#** | **Searches** | **Results** |
| --- | --- | --- |
| 1 | cleft palate/ or cleft lip/ or (((cleft or fissum) adj3 (palat$ or lip? or orofacial)) or (palate adj3 surg$) or palat?schi?is or harelip? or "hare lip" or cheiloschisis or labioschi?is).ti,ab,kf. | 29395 |
| 2 | exp "costs and cost analysis"/ or costs.tw. or cost effective$.tw. | 439101 |
| 3 | (Cost$ or cost benefit analys$ or health care costs).mp. | 684463 |
| 4 | 2 or 3 | 695616 |
| 5 | 1 and 4 | 374 |
| 6 | limit 5 to (english language and yr="2019 -Current") | 36 |

## EMBASE SEARCH

**Date:** Jan 21, 2019

**Search Strategy name:** "CLP and EconEval - EMBASE - FINAL" **Databases searched: Embase Classic+Embase** 1947 to 2019 January 18 **Search Strategy:**

| **#** | **Searches** | **Results** |
| --- | --- | --- |
| 1 | cleft palate/ or exp cleft lip/ or (((cleft or fissum) adj3 (palat$ or lip? or orofacial)) or (palate adj3 surg$) or palat?schi?is or harelip? or "hare lip" or cheiloschisis or labioschi?is).ti,ab,hw. | 36495 |
| 2 | (cost or costs).tw. | 654277 |
| 3 | 1 and 2 | 316 |
| 4 | limit 3 to (english language and yr="2000 -Current") | 271 |

**Update Date:** Feb 12, 2020

**Search Strategy name:** "CLP and EconEval - EMBASE - FINAL" Database(s): **Embase Classic+Embase** 1947 to 2020 February 11 Search Strategy:

| **#** | **Searches** | **Results** |
| --- | --- | --- |
| 1 | cleft palate/ or exp cleft lip/ or (((cleft or fissum) adj3 (palat$ or lip? or orofacial)) or (palate adj3 surg$) or palat?schi?is or harelip? or "hare lip" or cheiloschisis or labioschi?is).ti,ab,hw. | 38554 |
| 2 | (cost or costs).tw. | 725142 |
| 3 | 1 and 2 | 367 |
| 4 | limit 3 to (english language and yr="2019 -Current") | 39 |

### Cochrane Database of Systematic Reviews SEARCH

**Date:** Jan 25, 2019

**Search Strategy name:** "CLP and EconEval - COCHRANE - FINAL"

**Databases searched:** Cochrane Database of Systematic Reviews, Issue 1 of 12, January 2019 Date Run: 25/01/2019 21:54:53

| **ID** | **Search** | **Hits** |
| --- | --- | --- |
| #1 | MeSH descriptor: [Cleft Palate] explode all trees | 272 |
| #2 | MeSH descriptor: [Cleft Lip] explode all trees | 203 |
| #3 | (((cleft NEAR3 (palate or lip or orofacial)) or (palate NEAR3 surg*) or harelip)) (Word  variations have been searched) | 69 |
| #4 | #1 or #2 or #3 | 392 |
| #5 | MeSH descriptor: [Costs and Cost Analysis] explode all trees | 9578 |
| #6 | (costs OR cost effective*) | 42146 |
| #7 | ((Cost* or cost benefit analys* or health care costs)) | 60221 |
| #8 | #5 or #6 or #7 | 60252 |
| #9 | #4 and #8 with Cochrane Library publication date Between Jan 2000 and Jan 2019, in  Cochrane Reviews, Cochrane Protocols | 52 |

**Update Date:** Feb 12, 2020

**Search Name:** CLP and EconEval - COCHRANE - FINAL

**Date Run:** 12/02/2020 18:25:42

**Comment:** Karen Chung (kchung@qmed.ca), 25 Jan 2019

| **ID** | **Search** | **Hits** |
| --- | --- | --- |
| #1 | MeSH descriptor: [Cleft Palate] explode all trees | 277 |
| #2 | MeSH descriptor: [Cleft Lip] explode all trees | 211 |
| #3 | (((cleft NEAR3 (palate or lip or orofacial)) or (palate NEAR3 surg*) or harelip)) (Word  variations have been searched) | 79 |
| #4 | #1 or #2 or #3 | 417 |
| #5 | MeSH descriptor: [Costs and Cost Analysis] explode all trees | 10196 |
| #6 | (costs OR cost effective*) | 57352 |
| #7 | ((Cost* or cost benefit analys* or health care costs)) | 81463 |
| #8 | #5 or #6 or #7 | 81494 |
| #9 | #4 and #8 with Cochrane Library publication date Between Jan 2019 and Feb 2020, in  Cochrane Reviews, Cochrane Protocols | 5 |

**Global Index Medicus SEARCH Date:** Jan 22, 2019

### Databases searched:

Global Index Medicus – GIM; Regional Indexes:

AIM (AFRO), LILACS (AMRO/PAHO), IMEMR (EMRO), IMSEAR (SEARO), WPRIM (WPRO)

### Search Strategy:

| **Searches** | **Results** |
| --- | --- |
| (tw:(cleft palate OR cleft lip OR cleft palate surgery OR fissum OR orofacial OR palatoschizis OR palatischisis OR palatoschisis OR harelip OR harelips OR "hare lip" OR cheiloschisis OR labioschisis)) AND (tw:(cost OR costs)) AND (instance:"ghl") AND ( la:("en") AND ( year_cluster:("2012" OR "2015" OR "2007" OR "2008" OR "2013" OR "2002" OR "2003" OR "2004" OR "2006" OR "2009" OR "2016"  OR "2017" OR "2018")) | 21 |

| LILACS (Americas) (9) WPRIM (Western Pacific) (6) IMSEAR (South-EastAsia) (4)  IMEMR (Eastern Mediterranean) (2) | Korean (4)  Portuguese (3)  Spanish (1) |
| --- | --- |

**Update Date:** Feb 12, 2020 No results


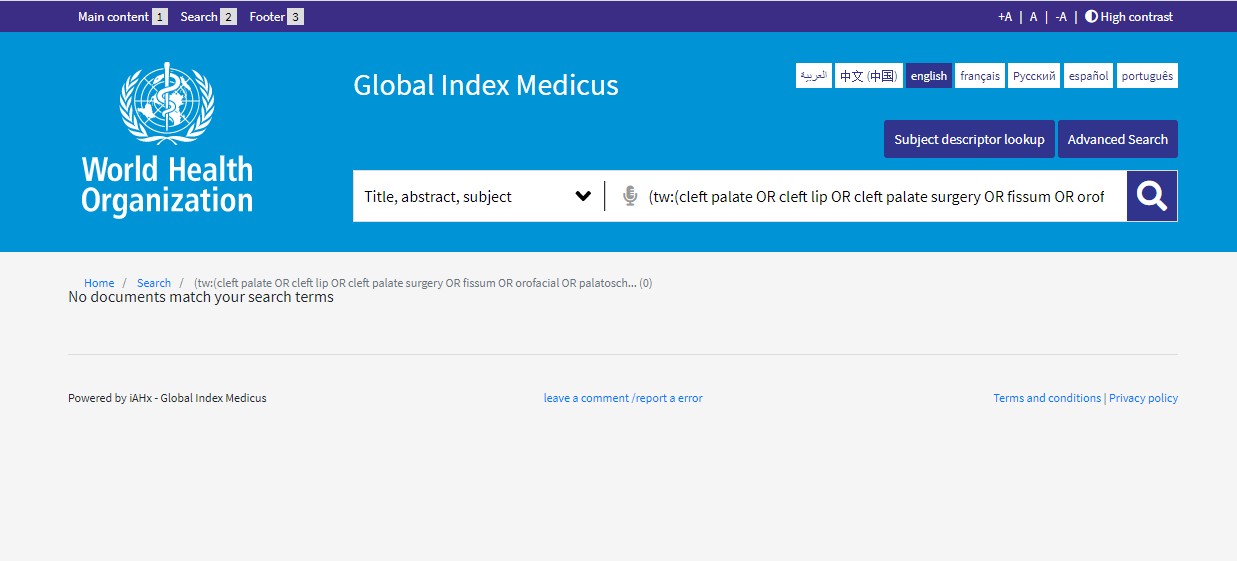


**CRD Databases SEARCH Date:** Jan 22, 2019

### Databases searched:

Centre for Reviews and Dissemination (CRD) Databases:

Health Technology Assessment Database (HTA) NHS Economic Evaluation Database (NHS EED) Database of Abstracts of Reviews of Effects (DARE)

**Search Strategy:** https://[www.crd.york.ac.uk/CRDWeb/HistoryPage.asp](http://www.crd.york.ac.uk/CRDWeb/HistoryPage.asp)


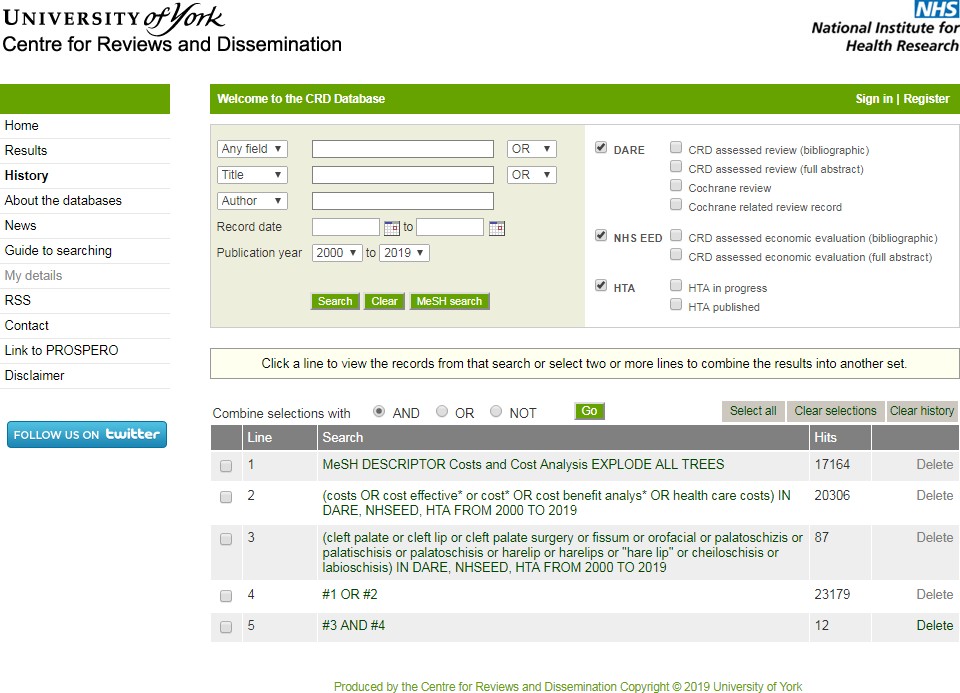


**Update Date:** Feb 12, 2020 No results


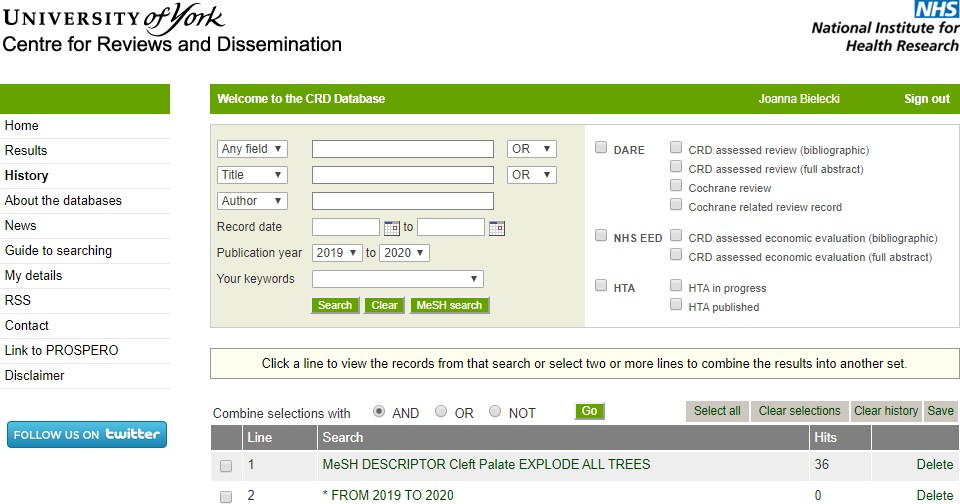


### HTA Database Canadian Repository SEARCH

**Date:** Jan 22, 2019

### Databases searched:

Centre for Reviews and Dissemination (CRD) Databases: HTA Database Canadian Repository

**Search Strategy:** <http://www.crd.york.ac.uk/PanHTA/HistoryPage.asp>


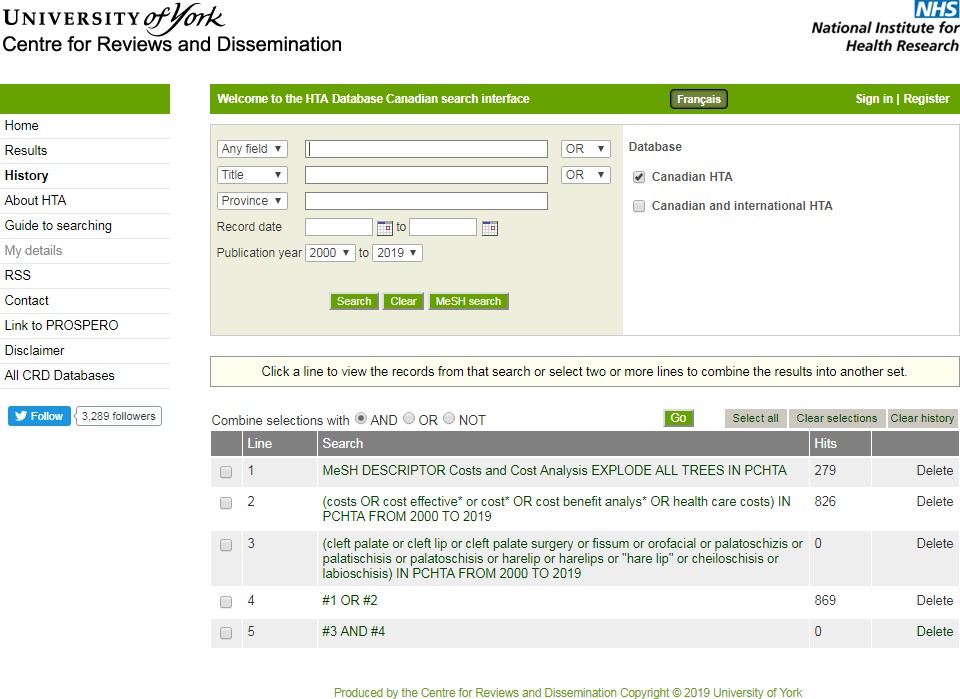


**Update Date:** Feb 12, 2020

This database is no longer available

### ScHARRHUD database SEARCH

**Date:** Jan 22, 2019

**Databases searched:** ScHARRHUD database

**Search Strategy:** 0 results for cleft palate or cleft lip


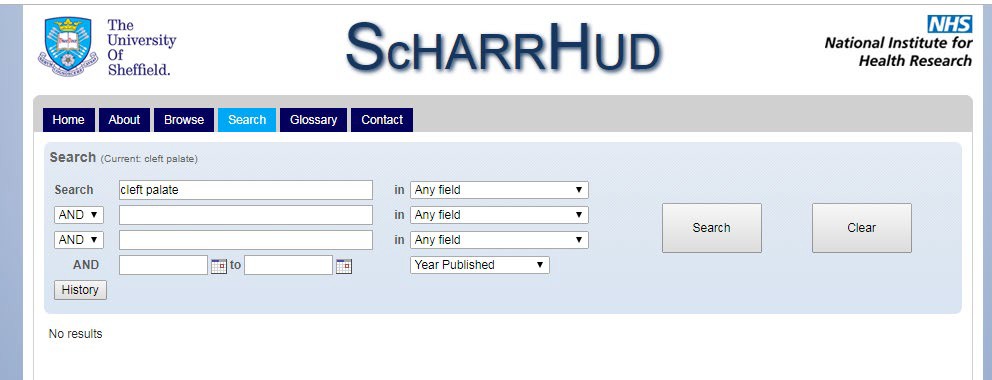


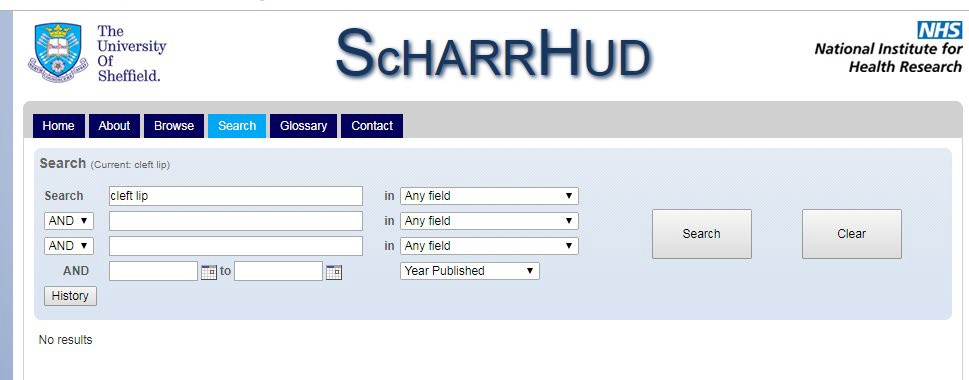


**Update Date:** Feb 12, 2020 No results


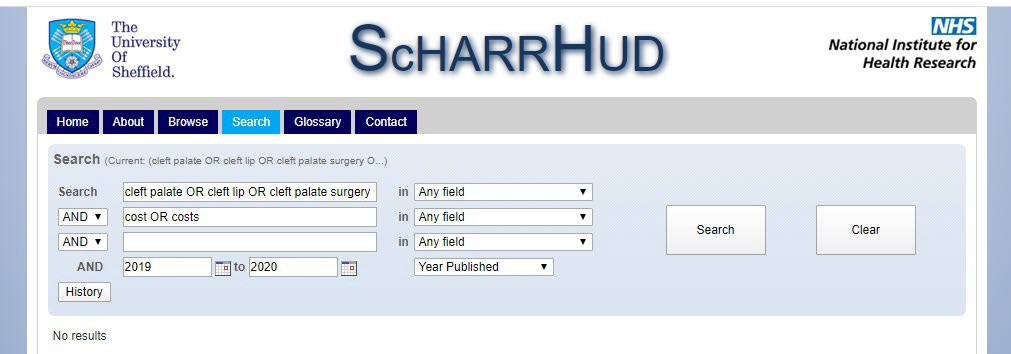


### CEA & GH CEA Registry SEARCH

**Date:** Jan 22, 2019

### Databases searched:

Global Health Cost Effectiveness Analysis (GH CEA) Registry Cost- Effectiveness Analysis (CEA) Registry

**Search Strategy:** 1 result – a duplicate from PubMed

(all the results below are from the same publication)


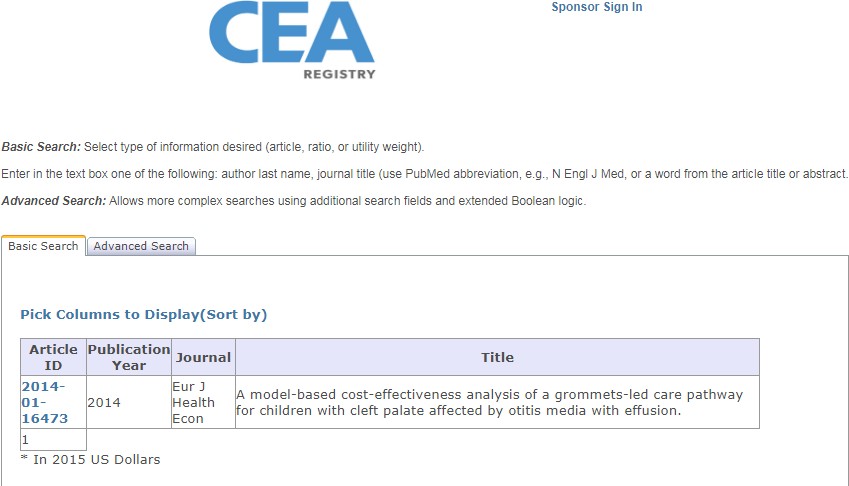


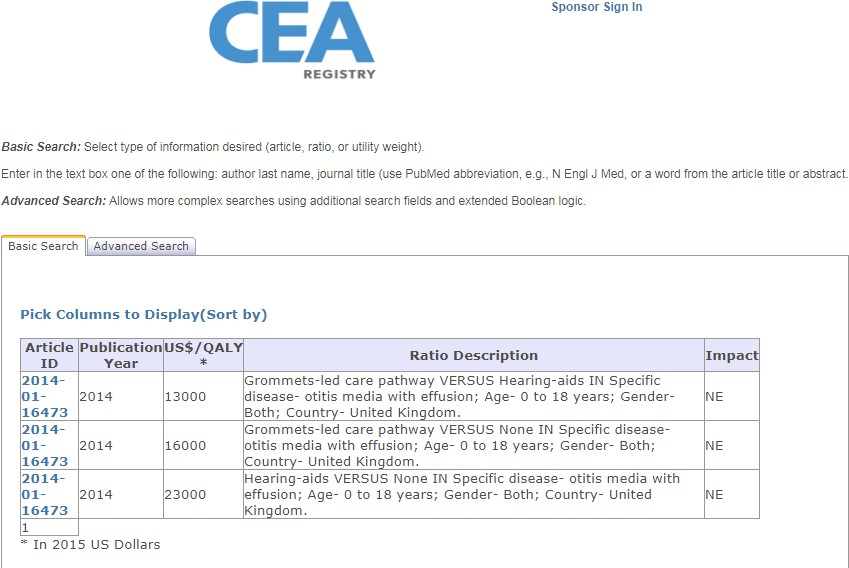


No results


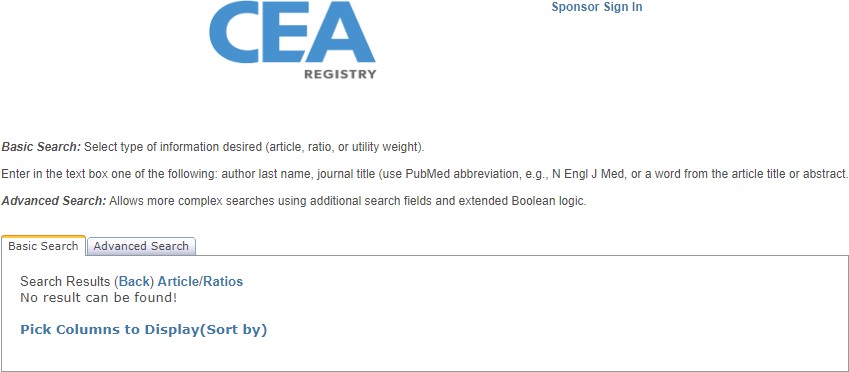


Duplicates removed by Endnote between databases (note: Endnote is not able to remove all duplicates due to indexing issues/differences so you should still expect some to be present):


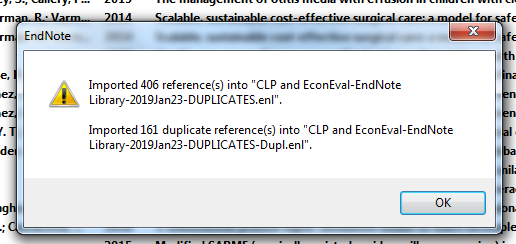

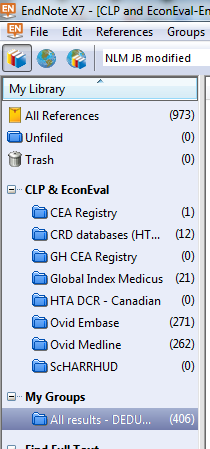


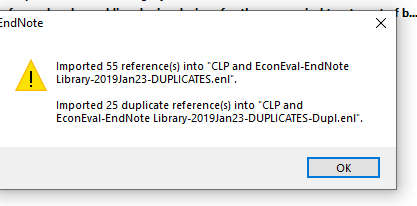


### Topic: The Cost-Effectiveness of Cleft Lip and/or Palate Surgery in Global Health: A Systematic Review

**Search done for:** Karen Chung (kchung@qmed.ca)

**Performed by:** Joanna Bielecki (joanna.bielecki@theta.utoronto.ca)

### Database(s) to be searched:

Ovid Medline (R) and Medline (R) In-Process and Other Non-Indexed Citations Ovid Embase

Global Index Medicus

Centre for Reviews and Dissemination (CRD) Databases: Health Technology Assessment Database (HTA) NHS Economic Evaluation Database (NHS EED) Database of Abstracts of Reviews of Effects (DARE) HTA Database Canadian Repository

ScHARRHUD database

Global Health Cost Effectiveness Analysis (GH CEA) Registry Cost- Effectiveness Analysis (CEA) Registry

Limits: 2000 to the present; English language

## MEDLINE SEARCH

**Date:** Jan 21, 2019

**Search Strategy name:** "CLP and EconEval - MEDLINE - FINAL"

### Databases searched:

Ovid MEDLINE: Epub Ahead of Print, In-Process & Other Non-Indexed Citations, Ovid MEDLINE® Daily and Ovid MEDLINE® 1946-Present

**Search Strategy:**

| **#** | **Searches** | **Results** |
| --- | --- | --- |
| 1 | Cleft Palate/ or Cleft Lip/ or ((Cleft adj3 (Palate? or lip? or Orofacial)) or (palate adj3 surg$) or Harelip?).ti,ab,kf. | 28119 |
| 2 | exp "costs and cost analysis"/ or costs.tw. or cost effective$.tw. | 409146 |
| 3 | (Cost$ or cost benefit analys$ or health care costs).mp. | 631666 |
| 4 | 2 or 3 | 642203 |
| 5 | 1 and 4 | 338 |
| 6 | limit 5 to (english language and yr="2000 -Current") | 261 |

## EMBASE SEARCH

**Date:** Jan 21, 2019

**Search Strategy name:** "CLP and EconEval - EMBASE - FINAL"

**Databases searched: Embase Classic+Embase** 1947 to 2019 January 18

### Search Strategy:

| **#** | **Searches** | **Results** |
| --- | --- | --- |
| 1 | cleft palate/ or exp cleft lip/ or (((cleft or fissum) adj3 (palat$ or lip? or orofacial)) or (palate  adj3 surg$) or palat?schi?is or harelip? or "hare lip" or cheiloschisis or labioschi?is).ti,ab,hw. | 36495 |
| 2 | (cost or costs).tw. | 654277 |
| 3 | 1 and 2 | 316 |
| 4 | limit 3 to (english language and yr="2000 -Current") | 271 |

**Global Index Medicus SEARCH Date:** Jan 22, 2019

### Databases searched:

Global Index Medicus – GIM; Regional Indexes:

AIM (AFRO), LILACS (AMRO/PAHO), IMEMR (EMRO), IMSEAR (SEARO), WPRIM (WPRO)

### Search Strategy:

| **Searches** | **Results** |
| --- | --- |
| (tw:(cleft palate OR cleft lip OR cleft palate surgery OR fissum OR orofacial OR palatoschizis OR palatischisis OR palatoschisis OR harelip OR harelips OR "hare lip" OR cheiloschisis OR labioschisis)) AND (tw:(cost OR costs)) AND (instance:"ghl") AND ( la:("en") AND ( year_cluster:("2012" OR "2015" OR "2007" OR "2008" OR "2013" OR "2002" OR "2003" OR  "2004" OR "2006" OR "2009" OR "2016" OR "2017" OR "2018")) | 21 |

| LILACS (Americas) (9) WPRIM (Western Pacific) (6) IMSEAR (South-EastAsia) (4)  IMEMR (Eastern Mediterranean) (2) | Korean (4)  Portuguese (3)  Spanish (1) |
| --- | --- |

**CRD Databases SEARCH**

**Date:** Jan 22, 2019

### Databases searched:

Centre for Reviews and Dissemination (CRD) Databases: Health Technology Assessment Database (HTA) NHS Economic Evaluation Database (NHS EED) Database of Abstracts of Reviews of Effects (DARE)

**Search Strategy:** https://[www.crd.york.ac.uk/CRDWeb/HistoryPage.asp](http://www.crd.york.ac.uk/CRDWeb/HistoryPage.asp)


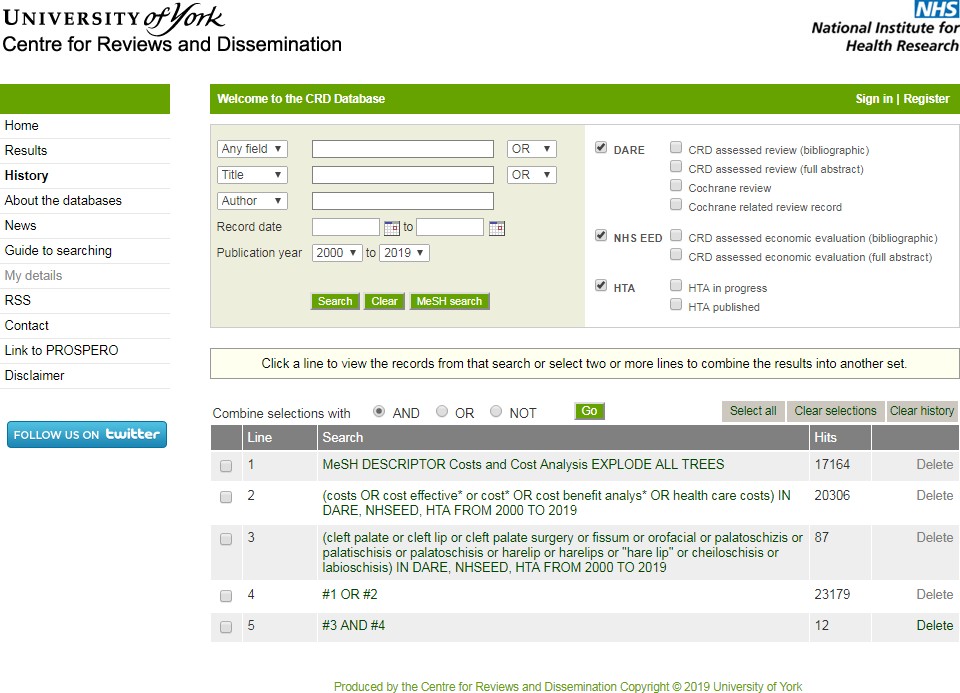


**HTA Database Canadian Repository SEARCH Date:** Jan 22, 2019

### Databases searched:

Centre for Reviews and Dissemination (CRD) Databases: HTA Database Canadian Repository

**Search Strategy:** <http://www.crd.york.ac.uk/PanHTA/HistoryPage.asp>


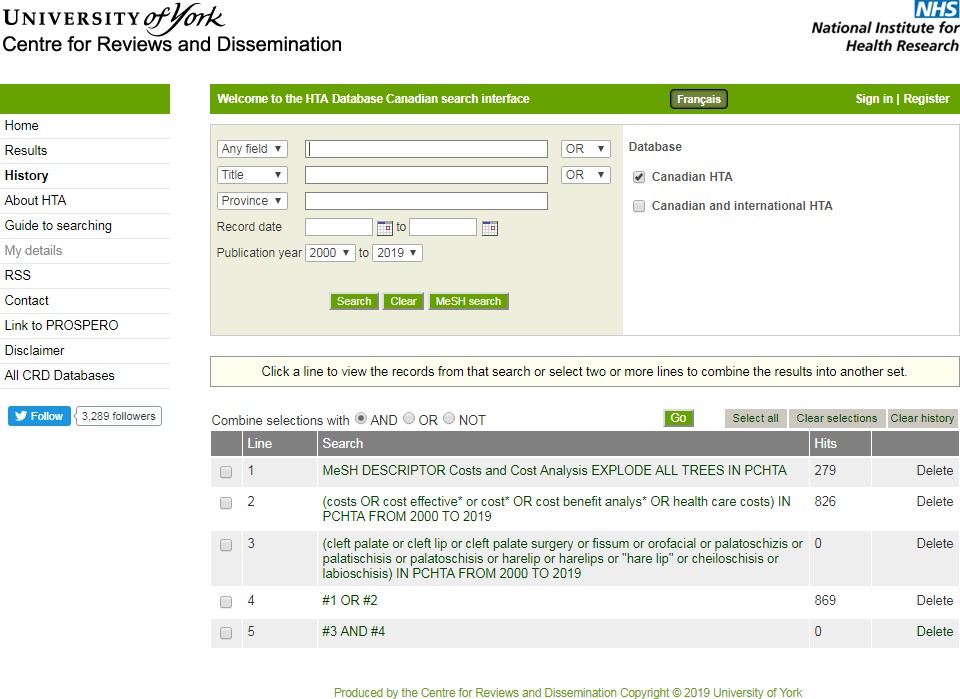


### ScHARRHUD database SEARCH

**Date:** Jan 22, 2019

**Databases searched:** ScHARRHUD database

**Search Strategy:** 0 results for cleft palate or cleft lip


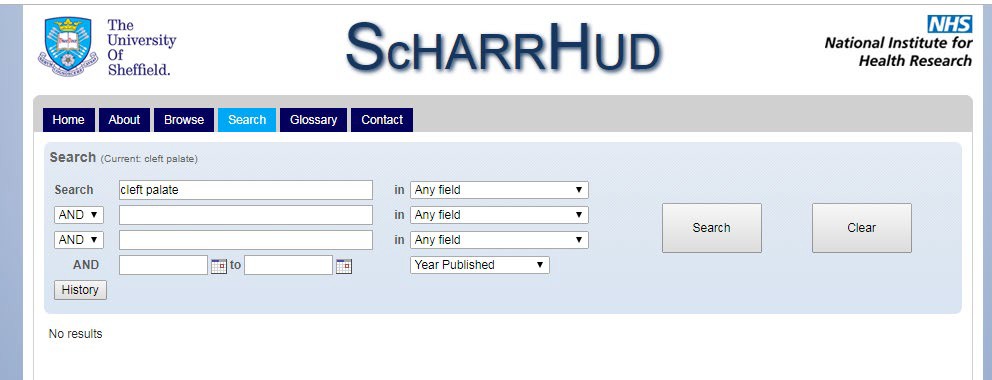


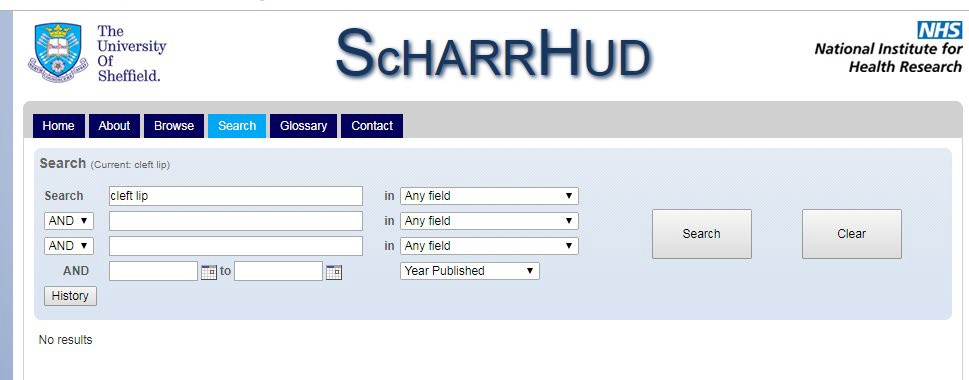


### CEA & GH CEA Registry SEARCH

**Date:** Jan 22, 2019

### Databases searched:

Global Health Cost Effectiveness Analysis (GH CEA) Registry Cost- Effectiveness Analysis (CEA) Registry

**Search Strategy:** 1 result – a duplicate from PubMed (all the results below are from the same publication)


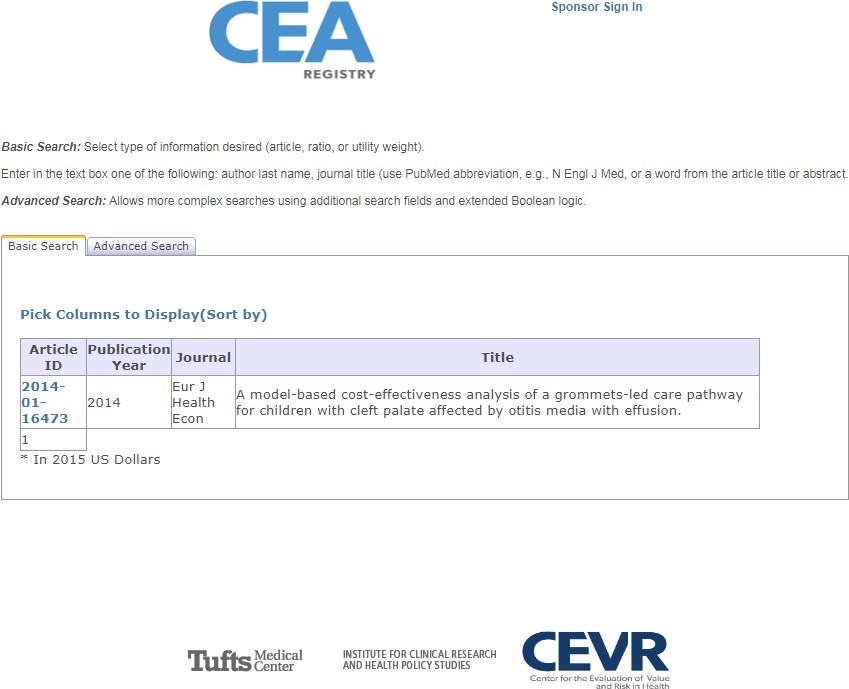


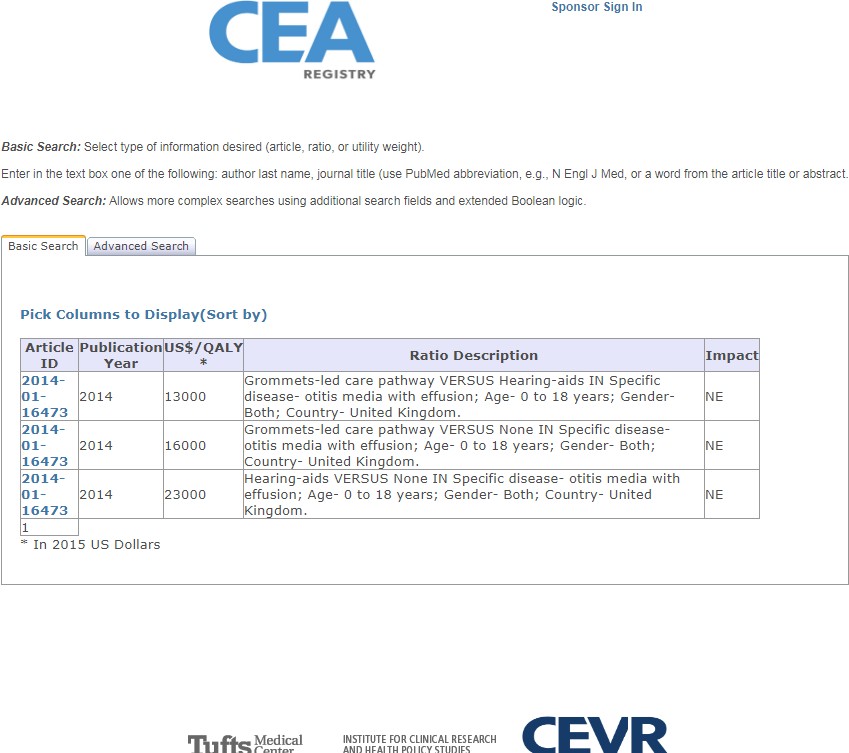


Duplicates removed by Endnote between databases (note: Endnote is not able to remove all duplicates due to indexing issues/differences so you should still expect some to be present):


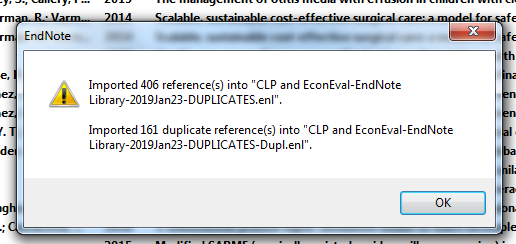

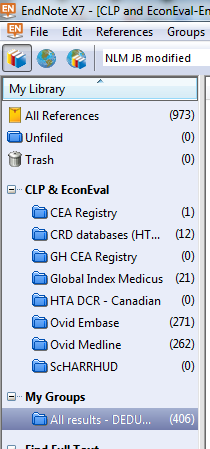

Supplement: sj-docx-1-cpc-10.1177_10556656221111028 - Supplemental material for A Systematic Review of the Cost-Effectiveness of Cleft Care in Low- and Middle-Income Countries: What is Needed? [file sj-docx-1-cpc-10.1177_10556656221111028.docx]
